# Nonmuscle Myosin Heavy Chain IIA Recognizes Sialic Acids on Sialylated RNA Viruses To Suppress Proinflammatory Responses via the DAP12-Syk Pathway

**DOI:** 10.1128/mBio.00574-19

**Published:** 2019-05-07

**Authors:** Yingqi Liu, Rui Li, Xin-xin Chen, Yubao Zhi, Ruiguang Deng, En-min Zhou, Songlin Qiao, Gaiping Zhang

**Affiliations:** a College of Veterinary Medicine, Northwest A&F University, Yangling, Shaanxi, China; b Key Laboratory of Animal Immunology of the Ministry of Agriculture, Henan Provincial Key Laboratory of Animal Immunology, Henan Academy of Agricultural Sciences, Zhengzhou, Henan, China; c College of Animal Science and Veterinary Medicine, Henan Agricultural University, Zhengzhou, Henan, China; Virginia Polytechnic Institute and State University

**Keywords:** DAP12, NMHC-IIA, negative modulation, proinflammatory responses, sialic acids, sialylated RNA virus

## Abstract

Viral infections induce proinflammatory signaling cascades and inflammatory cytokine production, which is precisely regulated for host benefits. In the current study, we unravel a previously unappreciated role of nonmuscle myosin heavy chain IIA (NMHC-IIA) as a negative regulator in inflammatory responses. We identified that cell surface NMHC-IIA recognized sialic acids on sialylated RNA viruses during early infections and interacted with an immune adaptor DNAX activation protein of 12 kDa (DAP12) to recruit downstream spleen tyrosine kinase (Syk), leading to suppressed virus-triggered proinflammatory responses. More importantly, recognition of sialylated RNA viruses or sialic acid mimics by NMHC-IIA was shown to inhibit lipopolysaccharide (LPS)-induced proinflammatory responses via the DAP12-Syk pathway. These findings uncover a novel negative regulation mechanism of proinflammatory responses and provide a molecular basis to design anti-inflammatory drugs.

## Abstract (precis)

NMHC-IIA, a subunit of nonmuscle myosin IIA (NM-IIA), takes part in diverse physiological processes, including cell movement, cell shape maintenance, and signal transduction. Recently, NMHC-IIA has been demonstrated to be a receptor or factor contributing to viral infections. Here, we identified that NMHC-IIA recognizes sialic acids on sialylated RNA viruses, vesicular stomatitis virus (VSV) and porcine reproductive and respiratory syndrome virus (PRRSV). Upon recognition, NMHC-IIA associates with the transmembrane region of DAP12 to recruit Syk. Activation of the DAP12-Syk pathway impairs the host antiviral proinflammatory cytokine production and signaling cascades. More importantly, sialic acid mimics and sialylated RNA viruses enable the antagonism of LPS-triggered proinflammatory responses through engaging the NMHC-IIA–DAP12-Syk pathway. These results actually support that NMHC-IIA is involved in negative modulation of the host innate immune system, which provides a molecular basis for prevention and control of the sialylated RNA viruses and treatment of inflammatory diseases.

## INTRODUCTION

Host innate immune defenses are of great importance against initial viral infections. Upon infection, viral pathogen-associated molecular patterns (PAMPs) are recognized by host pattern recognition receptors (PRRs) on immune cells, including macrophages and dendritic cells (DCs), to induce downstream signaling pathways (1). The active signaling pathways subsequently produce type I interferons (IFNs) and proinflammatory cytokines, such as tumor necrosis factor alpha (TNF-α), interleukin-6 (IL-6), IL-8, IL-1β, and other effectors (2). All these responses contribute to restraining viral infections, promoting infected cell clearance, and activating the host adaptive immune system. However, excessive host immune responses usually lead to individuals’ dysfunctions and disorders, thereby requiring a fine-tuned modulation by various negative regulators.

In particular, an appropriate production of proinflammatory cytokines induces acute inflammation to suppress viral replication and prevent other opportunistic pathogens (3). Once viral triggers disappear, proinflammatory responses are immediately terminated by different mechanisms (4). However, if viruses are not eliminated during acute inflammation, the chronic inflammatory state will be established with continuous production of inflammatory cytokines, which may be detrimental to hosts (5). For example, AIDS, with the symptom of sustained inflammation, is caused by HIV persistent infection (6). In some cases, overproduction of proinflammatory cytokines, namely, cytokine storm, can be life-threatening, e.g., lethal viral septic shock (7). Consequently, antiviral proinflammatory responses are normally under precise control to achieve an efficient clearance of invading viruses and avoid immune damage.

Proinflammatory signaling cascades converge on the activation of nuclear factor kappa-light-chain-enhancer of activated B cells (NF-κB) as well as the mitogen-activated protein kinase (MAPK) family members, p38 MAPK, c-Jun N-terminal kinase (JNK), and extracellular signal-regulated kinase 1/2 (ERK1/2). NF-κB activation is characterized by degradation of the phosphorylated inhibitory protein IκBα and translocation of phosphorylated p65/p50 dimers to the nuclei, whereas MAPKs are activated with phosphorylation (8, 9). Their activation eventually increases the transcription of various proinflammatory cytokines. These proinflammatory signaling pathways are therefore often hijacked by viruses to establish infections or targeted by anti-inflammation modulators to prevent uncontrolled proinflammatory responses (10, 11).

Nonmuscle myosin heavy chain IIA (NMHC-IIA) participates in a variety of cellular physiological processes, such as cell contractility, shape maintenance, and signal transduction (12). In addition, cell surface NMHC-IIA has been reported to facilitate viral infections (13, 14). Here, we revealed a novel mechanism of negative regulation of host proinflammatory responses, where NMHC-IIA recognizes sialic acids on the sialylated RNA viruses or sialic acid mimics to suppress proinflammatory responses through the DAP12-Syk pathway.

## RESULTS

### The DAP12-Syk pathway suppresses PRRSV-triggered proinflammatory responses.

**FIG 1:**
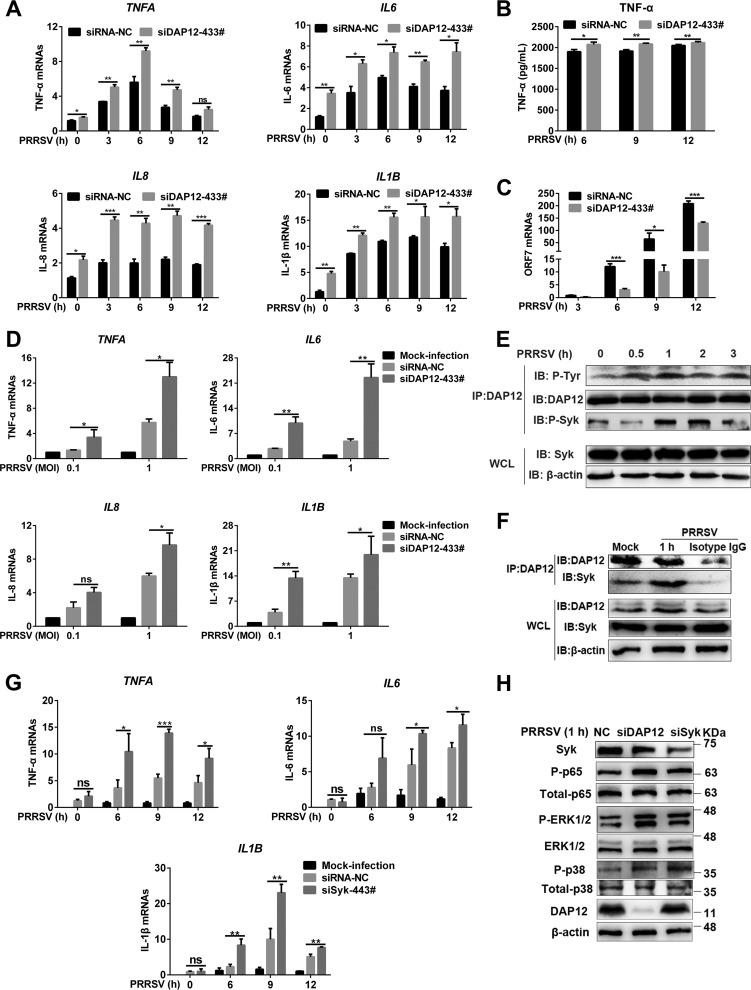
DAP12-Syk axis is activated to inhibit PRRSV-triggered proinflammatory responses. (A to C) *DAP12* knockdown enhances PRRSV-triggered production of proinflammatory cytokines and restricts PRRSV infection. PAMs were transfected with siDAP12-433# or siRNA-NC for 36 h and infected with PPRSV (MOI = 0.1) for indicated time periods (0, 3, 6, 9, and 12 h). “0 h” indicated that PRRSV was added at this time point and washed off immediately. qRT-PCR was used to measure proinflammatory cytokine (TNF-α, IL-6, IL-8, and IL-1β) mRNAs (A) and PRRSV ORF7 (C). TNF-α production was measured by ELISA (B). (D) *DAP12* knockdown promotes PRRSV-induced transcription of proinflammatory cytokines at different MOIs. PAMs with *DAP12* knockdown were infected by PRRSV (MOI = 0.1 or 1) for 6 h. qRT-PCR was performed to detect mRNA abundance of TNF-α, IL-6, IL-8, and IL-1β. (E) PRRSV early infection induces phosphorylation of DAP12 and Syk. PAMs were infected with PRRSV (MOI = 1) for indicated time periods (0, 0.5, 1, 2, and 3 h). DAP12 was immunoprecipitated by anti-DAP12 antibody. Phosphorylated DAP12 and Syk were detected by IB. (F) PRRSV infection enhances the association of DAP12 and Syk. PAMs were infected with PRRSV for 1 h. IB was conducted for DAP12-immunoprecipitated protein detection. (G) *Syk* knockdown promotes PRRSV-induced proinflammatory cytokine transcription. PAMs were transfected with siSyk-443# or siRNA-NC for 36 h and then infected with PRRSV (MOI = 0.1) for indicated time periods (0, 6, 9, and 12 h). *TNFA*, *IL-6*, and *IL1B* transcription was detected by qRT-PCR. (H) *DAP12* or *Syk* knockdown promotes PRRSV-induced phosphorylation of NF-κB, p38, and ERK1/2. *DAP12* or *Syk* knockdown PAMs were infected with PRRSV (MOI = 1) for 1 h. IB analysis was performed with indicated antibodies. Experiments in all panels were repeated at least three times, and similar results were obtained. Quantitation data were shown as mean ± SD from three replicates. Statistical analysis used in qRT-PCR was determined by Student’s *t* test: *, *P* < 0.05; **, *P* < 0.01; ***, *P* < 0.001; ns, not significant.

The economically critical *Arterivirus* porcine reproductive and respiratory syndrome virus (PRRSV) has been extensively studied on modulation of proinflammatory responses (15). DAP12, an immune adaptor, has been reported to be involved in regulation of virus-triggered immune responses (16). Initially, we tried to determine whether DAP12 regulated PRRSV-induced inflammatory cytokine production in its primary *in vivo* target, pulmonary alveolar macrophages (PAMs), with knockdown assays (see Fig. S1A and B in the supplemental material). The results showed that PRRSV infection induced transcription of proinflammatory cytokines (TNF-α, IL-6, IL-8, and IL-1β) (Fig. 1A) and production of TNF-α (Fig. 1B), but not for anti-inflammatory cytokine IL-10 (Fig. S1C). In contrast, *DAP12* knockdown promoted PRRSV-induced production of proinflammatory cytokines (Fig. 1A and B), while suppressing PRRSV infection as shown by decreased viral open reading frame (ORF) 7 replication (Fig. 1C) and nucleocapsid (N) expression (Fig. S1D). *DAP12* knockdown (Fig. S1E and F) also intensified PRRSV-induced proinflammatory cytokine transcription at different multiplicities of infection (MOIs) (Fig. 1D) and inhibited viral replication (Fig. S1G). Next, we explored whether DAP12 overexpression inhibited PRRSV-induced inflammatory cytokine production in the continuous PAM cell line CRL-2843-CD163 with undetectable endogenous DAP12 (Fig. S2A and B). DAP12 overexpression suppressed production of proinflammatory cytokines (Fig. S2C to E) and facilitated viral replication at different MOIs (Fig. S2F). These results indicated that DAP12 antagonized PRRSV-triggered production of proinflammatory cytokines.

10.1128/mBio.00574-19.1 FIG S1 Knockdown of DAP12 promotes PRRSV-triggered transcription of pro-inflammatory cytokines. (A and B) *DAP12* knockdown. Knockdown of DAP12 was determined by IB (A) and qRT-PCR (B) in PAMs infected with PRRSV (MOI = 0.1) for indicated time periods (0, 3, 6, 9, 12, 24, 36, and 48 h). (C) PRRSV infection does not induce transcription of anti-inflammatory cytokine IL-10. *IL10* transcription was detected by qRT-PCR in PAMs infected with PRRSV. (D) *DAP12* knockdown restricts PRRSV infection. PAMs were transfected with siDAP12-433# or siRNA-NC for 36 h and infected by PPRSV (MOI = 0.1) for 36 h. IB was conducted to determine N expression. (E to G) *DAP12* knockdown promotes PRRSV-induced transcription of proinflammatory cytokines and restrains PRRSV infection at different MOIs. PAMs with *DAP12* knockdown were infected by PRRSV (MOI = 0.1 or 1) for 6 h. *DAP12* knockdown efficiencies were determined by qRT-PCR (E) and IB where Image J software was used to analyze the relative levels of DAP12, where β-actin served as a loading control (F). PRRSV ORF7 was measured by qRT-PCR (G). Experiments in all panels were repeated at least three times, and similar results were obtained. Quantitation data are mean ± SD (*n* = 3). Statistical analysis used in qRT-PCR was performed using the Student *t* test: *, *P* < 0.05; **, *P* < 0.01; ***, *P* < 0.001. Download FIG S1, TIF file, 1.4 MB. Copyright © 2019 Liu et al. 2019 Liu et al. This content is distributed under the terms of the Creative Commons Attribution 4.0 International license.

10.1128/mBio.00574-19.2 FIG S2 DAP12 overexpression inhibits PRRSV-triggered proinflammatory cytokine production. (A and B) DAP12 overexpression confirmed by IB. CRL-2843-CD163 cells were transfected with 3×Flag-DAP12 (1 μg) or empty vector for 36 and then infected with PRRSV (MOI = 5 or 10). DAP12 abundance was detected by IB at indicated time points (0, 3, 6, 9, 12, 24, and 36 h postinfection [hpi]). (C to E) DAP12 overexpression inhibits PRRSV-triggered proinflammatory cytokine production at different MOIs. CRL-2843-CD163 cells were transfected with 3×Flag-DAP12 (1 μg) and then infected with PRRSV (MOI = 5 or 10). qRT-PCR was performed to detect *TNFA*, *IL6*, and *IL1B* transcription at indicated time points (0, 3, 6, 9, 12, 24, and 36 hpi) (C and D). Production of TNF-α was also detected by ELISA at 36 and 48 hpi (MOI = 5) (E). (F) DAP12 overexpression facilitates PRRSV replication at different MOIs. CRL-2843-CD163 cells were transfected with 3×Flag-DAP12 (1 μg) or empty vector and infected with PRRSV (MOI = 5 or 10). qRT-PCR was performed to detect PRRSV ORF7 for indicated time periods (3, 6, 9, and 12 hpi). Experiments in all panels were repeated at least three times, and similar results were obtained. Quantitation data are mean ± SD (*n* = 3). Statistical analysis used in qRT-PCR was performed using the Student *t* test: *, *P* < 0.05; **, *P* < 0.01; ***, *P* < 0.001; ns, not significant. Download FIG S2, TIF file, 1.3 MB. Copyright © 2019 Liu et al. 2019 Liu et al. This content is distributed under the terms of the Creative Commons Attribution 4.0 International license.

We subsequently sought to determine the inhibitory mechanism of DAP12. Viral infections have been shown to induce tyrosine (Y) phosphorylation within the DAP12 immunoreceptor tyrosine-based activation motif (ITAM) and then recruit phosphorylated Syk (17). In this study, obvious phosphorylation of DAP12 and Syk was observed during PRRSV early infection (Fig. 1E). Immunoprecipitation (IP) assays indicated that DAP12 was constitutively associated with Syk, and the association was enhanced upon PRRSV infection in PAMs (Fig. 1F) and CRL-2843-CD163 cells (Fig. S3A). The interaction of these two proteins was also verified in the overexpression system of human embryonic kidney 293T (HEK-293T) cells (Fig. S3B and C). Y86 and Y97 within the DAP12 ITAM were further shown to be indispensable for its interaction with Syk, while aspartic acid (D) 50 within the DAP12 transmembrane domain (TMD) was dispensable (Fig. S3D).

10.1128/mBio.00574-19.3 FIG S3 DAP12 interacts with Syk. (A) PRRSV infection enhances the interaction of DAP12 and Syk. CRL-2843-CD163 cells were transfected with Syk-myc-his (6 μg) and 3×Flag-DAP12 (6 μg) and then infected by PRRSV (MOI = 1) for 1 h. WCLs were subjected to IP assay with anti-Flag MAb for detection of interaction between DAP12 and Syk. (B and C) DAP12 and Syk interact with each other in HEK-293T cells. Cells were cotransfected with 3×Flag-DAP12 (6 μg) and Syk-myc-his (6 μg) for 48 h. Co-IP assays with anti-Myc (B) or anti-Flag (C) MAb determined the interaction. (D) Y86 and Y97 within the DAP12 ITAM are indispensable for the interaction between DAP12 and Syk, while D50 within the DAP12 TMD is dispensable. CRL-2843-CD163 cells were transfected with 3×Flag-DAP12 (6 μg) or the indicated 3×Flag-DAP12-mutants (6 μg) for 48 h and then infected with PRRSV (MOI = 5) for 1 h. Flag-tagged proteins were immunoprecipitated and subjected to IB with indicated antibodies. Experiments in all panels were repeated at least three times, and similar results were obtained. Download FIG S3, TIF file, 2.6 MB. Copyright © 2019 Liu et al. 2019 Liu et al. This content is distributed under the terms of the Creative Commons Attribution 4.0 International license.

*Syk* knockdown (Fig. S4A and B) or noncytotoxic Syk inhibitor R406 (Fig. S4D) in PAMs significantly promoted PRRSV-induced proinflammatory cytokine transcription and restrained PRRSV infection as *DAP12* knockdown did (Fig. 1G and Fig. S4C, E, and F). Additionally, DAP12 overexpression failed to inhibit the transcription of proinflammatory cytokines in *Syk* knockdown CRL-2843-CD163 cells, resulting in the decreased PRRSV replication (Fig. S4G to I). These results illustrated that the DAP12-Syk pathway was involved in antagonism of PRRSV-triggered proinflammatory cytokine production.

10.1128/mBio.00574-19.4 FIG S4 *Syk* knockdown or inhibition promotes transcription of proinflammatory cytokines and suppresses PRRSV infection. (A to C) *Syk* knockdown inhibits PRRSV infection. PAMs were transfected with siSyk-443# or siRNA-NC for 36 h and then infected with PRRSV (MOI = 0.1) for indicated time periods (6, 9, and 12 h). *Syk* knockdown was confirmed by qRT-PCR (A) and IB (B). PRRSV ORF7 was measured by qRT-PCR (C). (D) Treatment with R406 (5 μM) has no cytotoxicity. Cell viability was measured in PAMs treated with 5 μM R406 for 12 h. (E and F) Inhibition of Syk promotes proinflammatory cytokine transcription and suppresses PRRSV infection. PAMs were treated with 5 μM R406 for 6 h and then infected by PRRSV (MOI = 0.1) for indicated time periods (0, 6, 9, and 12 h). qRT-PCR was performed to detect mRNA abundance of TNF-α, IL-6, and IL-1β (E) and PRRSV ORF7 (F). (G to I) After *Syk* knockdown, DAP12 overexpression fails to inhibit PRRSV-induced proinflammatory cytokine transcription and promote PRRSV replication. CRL-2843-CD163 cells with DAP12 overexpression were transfected with siSyk-443# for 36 h and infected by PPRSV (MOI = 10) for indicated time periods (0, 6, and 12 h). qRT-PCR was performed to detect mRNA abundance of TNF-α, IL-6, and IL-1β (G) and PRRSV ORF7 (H). IB was adopted to evaluate the abundance of DAP12 and Syk (I). Experiments in all panels were repeated at least three times, and similar results were obtained. Quantitation data are mean ± SD (*n* = 3). Statistical analysis used in qRT-PCR was performed using the Student *t* test: *, *P* < 0.05; **, *P* < 0.01; ***, *P* < 0.001; ns, not significant. Download FIG S4, TIF file, 1.4 MB. Copyright © 2019 Liu et al. 2019 Liu et al. This content is distributed under the terms of the Creative Commons Attribution 4.0 International license.

To determine which proinflammatory cascades were targeted by the DAP12-Syk pathway, we used noncytotoxic inhibitors of NF-κB and MAPKs to treat PAMs and then inoculated PAMs with PRRSV (Fig. S5A). We observed that PRRSV-induced proinflammatory cytokine transcription was mediated by activation of NF-κB and MAPK (p38 and ERK1/2) pathways (Fig. S5B and C). *DAP12* or *Syk* knockdown enforced these activations in response to PRRSV (Fig. 1H). In CRL-2843-CD163 cells, DAP12 overexpression repressed PRRSV-triggered NF-κB activation and proinflammatory cytokine transcription, while *Syk* knockdown restored the proinflammatory responses (Fig. S5D to F). Phosphorylated p38 and ERK1/2 were hardly detected in CRL-2843-CD163 cells (data not shown). The results demonstrated that the DAP12-Syk pathway suppressed MAPK (ERK1/2, p38)- and NF-κB-mediated proinflammatory responses triggered by PRRSV.

10.1128/mBio.00574-19.5 FIG S5 DAP12-Syk axis inhibits NF-κB and MAPK (p38 and ERK1/2) activation during PRRSV early infection. (A) The noncytotoxic concentrations of specific inhibitors were determined by cell viability assay. PAMs were treated with increasing doses (0, 5, 10, 15, 20, 25, and 30 μM) of indicated inhibitors for 12 h. Cell viabilities were measured. (B) NF-κB and MAPK (p38 and ERK1/2) pathways are involved in PRRSV-induced proinflammatory cytokine transcription. PAMs were treated with indicated nontoxic inhibitors for 11 h and later infected with PRRSV (MOI = 0.1) for 1 h. Transcription of TNF-α, IL-6, and IL-8 was determined by qRT-PCR. (C) NF-κB and MAPKs (p38 and ERK1/2) are activated during PRRSV early infection. PAMs were infected with PRRSV (MOI = 1) for 1 h. IB was performed to determine the abundance of phosphorylated p65 and MAPKs (p38 and ERK1/2). (D to F) *Syk* knockdown recovers the attenuated proinflammatory responses by DAP12 overexpression. CRL-2843-CD163 cells were cotransfected with 3×Flag-DAP12 (1 μg) and siSyk-443# for 36 h and then infected by PRRSV (MOI = 10) for indicated time periods (0, 1, 3, and 6 h). Phosphorylated p65 was detected by IB (D). qRT-PCR was used to measure *Syk* (E) and *TNFA*, *IL6*, *IL8*, and *IL1B* (F) mRNAs. Experiments in all panels were repeated at least three times, and similar results were obtained. Quantitation data are mean ± SD (*n* = 3). Statistical analysis used in qRT-PCR was performed using the Student *t* test: *, *P* < 0.05; **, *P* < 0.01; ns, not significant. Download FIG S5, TIF file, 2.1 MB. Copyright © 2019 Liu et al. 2019 Liu et al. This content is distributed under the terms of the Creative Commons Attribution 4.0 International license.

### NMHC-IIA is identified to interact with DAP12.

**FIG 2:**
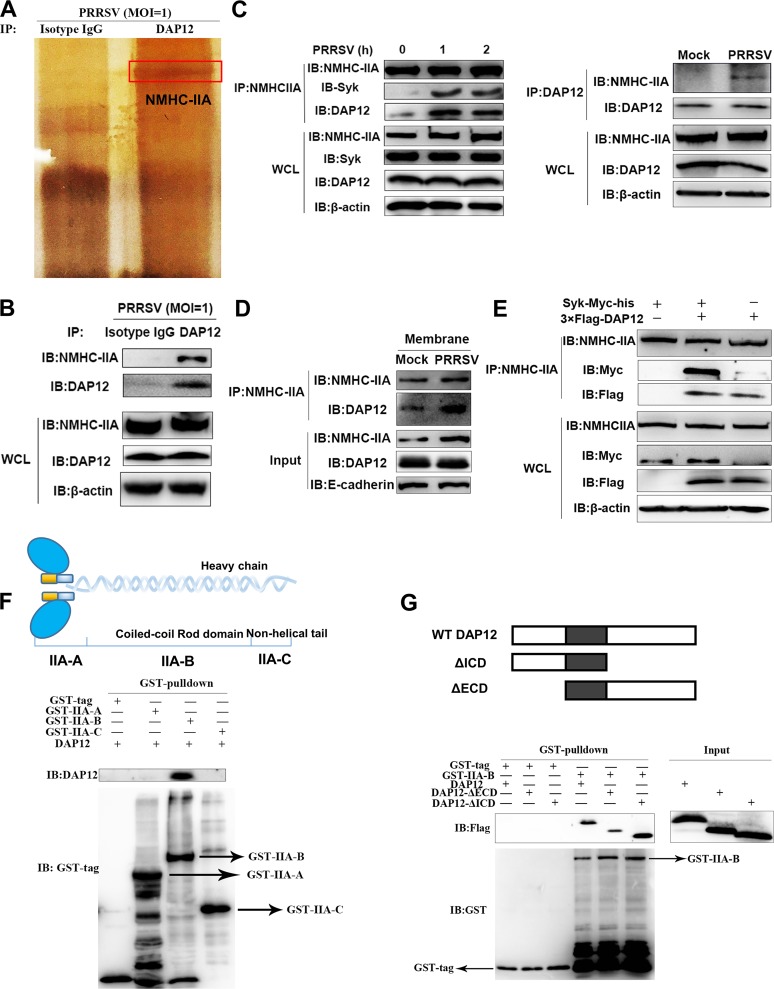
NMHC-IIA interacts with DAP12. (A and B) NMHC-IIA is identified to be a DAP12-associated protein. PAMs were infected with PRRSV (MOI = 1) for 1 h. DAP12 was immunoprecipitated with anti-DAP12 MAb, where isotype IgG served as a control. The eluted proteins were then subjected to SDS-PAGE using silver staining followed by MS (A) or IB analysis (B). (C) The interaction of NMHC-IIA and DAP12 is enhanced upon PRRSV infection. PAMs were infected with PRRSV (MOI = 1) for indicated time periods (0, 1, and 2 h). IP assays with anti-DAP12 or anti-NMHC-IIA MAb were conducted to determine their association. (D) NMHC-IIA interacts with DAP12 on the cell membranes. NMHC-IIA was immunoprecipitated from membrane proteins of PRRSV-infected PAMs. The proteins were then detected by IB with the indicated antibodies. (E) NMHC-IIA specifically interacts with DAP12. CRL-2843-CD163 cells were transfected with 3×Flag-DAP12 or Syk-myc-his for 48 h. IB was performed to detect NMHC-IIA-immunoprecipitated proteins. (F) IIA-B is required for its interaction with DAP12. After incubation with GST-tagged NMHC-IIA fragments (IIA-A, IIA-B, and II-C), GST resins were incubated with the purified DAP12. The proteins were eluted and applied to IB analysis. (G) DAP12 TMD is required for its interaction with IIA-B. GST resins were incubated with purified GST-IIA-B and with the purified DAP12 or DAP12 truncations (DAP12-ΔICD or DAP12-ΔECD). The proteins were eluted and applied to IB analysis. Experiments in all panels were repeated at least three times, and similar results were obtained.

In general, association of a receptor with DAP12 is responsible for activating the DAP12-Syk pathway (18). Here, we carried out IP assays followed by mass spectrometry (MS) analysis to screen potential DAP12-associated receptors. Among the MS-identified proteins, NMHC-IIA was one of the most prominent proteins binding to DAP12 (Fig. 2A). The binding of NMHC-IIA to DAP12 was also confirmed by immunoblotting (IB) analysis (Fig. 2B). We further found that the association of endogenous NMHC-IIA and DAP12 was augmented upon PRRSV infection (Fig. 2C). Moreover, localization of NMHC-IIA and DAP12 was visualized in CRL-2843-CD163 cells cotransfected with enhanced green fluorescent protein (EGFP)-NMHC-IIA and DAP12-monomeric red fluorescent protein (mRFP). Though NMHC-IIA and DAP12 sparsely colocalized in the cell membranes, their colocalization was increased after PRRSV infection (Fig. S6A). We also immunoprecipitated NMHC-IIA from membrane proteins of PRRSV-infected PAMs. The results of immunoblotting (IB) analysis showed that PRRSV infection intensified the association of NMHC-IIA and DAP12 on the cell surface (Fig. 2D). We further observed that NMHC-IIA specifically interacted with DAP12 but not with Syk in CRL-2843-CD163 cells transfected with 3×Flag-DAP12 and Syk-myc-His (Fig. 2E). Additional evidence from immunofluorescence assay (IFA) indicated that punctately distributed NMHC-IIA was located at the cell membranes where DAP12 colocalized with Syk in mock-infected cells, while PRRSV infection increased their colocalization (Fig. S6B). Collectively, NMHC-IIA was identified to be a novel binding protein for DAP12.

10.1128/mBio.00574-19.6 FIG S6 PRRSV infection enhances colocalization of NMHC-IIA, DAP12, and Syk. (A) PRRSV infection increases the colocalization of NMHC-IIA and DAP12. CRL-2843-CD163 cells were transfected with EGFP-NMHC-IIA (green) and DAP12-mRFP (red) for 36 h and infected or mock infected with PRRSV (MOI = 1) for 1 h. The cells were fixed with 4% paraformaldehyde followed by confocal microscopy. Bars, 10 μm. Shown are the representative images. (B) Colocalization of NMHC-IIA, DAP12, and Syk was increased upon PRRSV infection. CRL-2843-CD163 cells were cotransfected with EGFP-NMHC-IIA (green) and DAP12-mRFP (red) for 36 h and later infected with PRRSV for 1 h. The cells were fixed and stained for Syk (blue) by confocal microscopy. Bars, 10 μm. Shown are representative images. Confocal microscopy experiments were repeated three times, and similar results were obtained. Download FIG S6, TIF file, 2.9 MB. Copyright © 2019 Liu et al. 2019 Liu et al. This content is distributed under the terms of the Creative Commons Attribution 4.0 International license.

Next, we explored how NMHC-IIA interacted with DAP12. On one hand, NMHC-IIA was divided into three fragments from N to C terminus, denominated IIA-A (residues 1 to 742; the numbering is according to UniProt entry F1SKJ1), containing myosin N-terminal SRC homology 3 domain (SH3)-like domain and actin-binding domain; IIA-B (residues 743 to 1560), containing the coiled-coil rod domain; and IIA-C (residues 1561 to 1957), with the nonhelical tail. Glutathione *S*-transferase (GST) pulldown assays showed that IIA-B was responsible for the interaction with DAP12 (Fig. 2F). IP assay further indicated that the interaction was independent of D50 and the five Y’s in DAP12 (Fig. S7A). On the other hand, DAP12 was separated into two parts, ΔICD with deletion of the intracellular domain (ICD) and ΔECD with deletion of the extracellular domain (ECD). Pulldown assays determined that both ΔICD and ΔECD bound to IIA-B, suggesting that the DAP12 TMD might be the binding region (Fig. 2G and Fig. S7B). Furthermore, a short stretch (residues 51 to 57; the numbering is according to UniProt entry Q9TU45) within the DAP12 TMD was proven to be indispensable for the interaction (Fig. S7C and D).

10.1128/mBio.00574-19.7 FIG S7 Interaction of DAP12 with NMHC-IIA depends on its TMD. (A) The association of NMHC-IIA with DAP12 is not required with D50 and Y’s of DAP12. CRL-2843-CD163 cells were transfected with 3×Flag-DAP12 or the indicated 3×Flag-DAP12-mutants (6 μg). NMHC-IIA was immunoprecipitated from WCLs. IB was performed with the indicated antibodies. (B) The DAP12 TMD is required for its interaction with IIA-B. Protein A/G beads were incubated with purified Fc-IIA-B at 4°C for 2 h and then subjected to secondary incubation with purified DAP12, DAP12-ΔICD, or DAP12-ΔECD at 4°C for 30 min. The proteins were eluted and applied to IB analysis. (C and D) Residues 50 to 57 within DAP12 TMD are indispensable for its interaction with IIA-B. GST-resins or protein A/G beads were incubated with purified GST-IIA-B (C) or Fc-IIA-B (D) at 4°C for 2 h and then with the purified DAP12 truncations (DAP12-ΔTM1, -ΔTM2, or -ΔTM3) at 4°C for 30 min. The proteins were eluted and applied to IB analysis. Experiments in all panels were repeated at least three times, and similar results were obtained. Download FIG S7, TIF file, 2.9 MB. Copyright © 2019 Liu et al. 2019 Liu et al. This content is distributed under the terms of the Creative Commons Attribution 4.0 International license.

### The NMHC-IIA–DAP12-Syk pathway is involved in inhibition of PRRSV-triggered proinflammatory responses.

**FIG 3:**
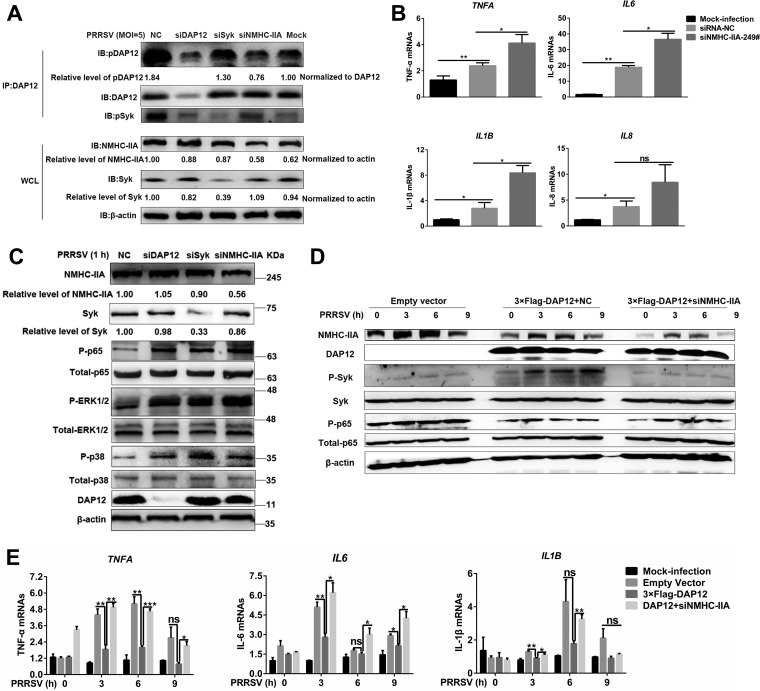
NMHC-IIA-DAP12-Syk is involved in inhibition of PRRSV-triggered proinflammatory responses. (A) NMHC-IIA is upstream of the DAP12-Syk pathway. PAMs were transfected with siNMHC-IIA-249#, siDAP12, or siSyk for 48 h and infected by PRRSV (MOI = 1) for 1 h. DAP12 was immunoprecipitated with anti-DAP12 MAb. IB was performed with specific antibodies. (B and C) Knockdown of NMHC-IIA promotes proinflammatory responses. PAMs were mock infected or infected with PRRSV (MOI = 1) for 1 h after transfection with siRNA-NC or siNMHC-IIA for 48 h. qRT-PCR was used to detect *TNFA*, *IL-6*, *IL-8*, and *IL1B* transcription (B). IB was performed to detect phosphorylated NF-κB and MAPKs (ERK1/2 and p38) (C). (D and E) NMHC-IIA inhibits PRRSV-induced proinflammatory responses. CRL-2843-CD163 cells were transfected with 3×Flag-DAP12 (1 μg) and siNMHC-IIA or siRNA-NC for 48 h and then infected with PRRSV (MOI = 5) for indicated periods (0, 3, 6, and 9 h). IB was performed to detect indicated proteins in the NF-κB pathway (D). qRT-PCR was used to detect *TNFA*, *IL-6*, and *IL1B* transcription (E). Experiments in all panels were repeated at least three times, and similar results were obtained. Quantitation shown was from three independent experiments (mean ± SD from three replicates). Statistical analysis used in qRT-PCR was performed using Student’s *t* test: *, *P* < 0.05; **, *P* < 0.01; ***, *P* < 0.001; ns, not significant.

To determine whether NMHC-IIA was upstream of the DAP12-Syk pathway, we examined PRRSV-induced phosphorylation of DAP12 and Syk in *myosin heavy chain 9* (*MYH9*; encoding NMHC-IIA), *DAP12*, or *Syk* knockdown cells. Strikingly, more than 70% *MYH9* knockdown efficiency caused significant cell damage (Fig. S8A). We found that *DAP12* knockdown weakened phosphorylation of Syk, but noncytotoxic *MYH9* knockdown (about 50% efficiency) impaired phosphorylation of DAP12 and Syk in PAMs (Fig. 3A) and CRL-2843-CD163 cells (Fig. S8B). Additionally, blebbistatin, a specific inhibitor of NMHC-IIA activity (19), suppressed phosphorylation of DAP12 and Syk (Fig. S8C). All these results demonstrated that NMHC-IIA was upstream of DAP12 and required for activation of the DAP12-Syk pathway.

10.1128/mBio.00574-19.8 FIG S8 The NMHC-IIA-DAP12-Syk pathway suppresses PRRSV-induced proinflammatory responses. (A) Knockdown of NMHC-IIA. PAMs were transfected with siRNA-NC or siNMHC-IIA-249# (10 and 20 nM) for 48 h. Cell viability and NMHC-IIA abundance were measured, respectively. (B and C) NMHC-IIA is responsible for activation of the DAP12-Syk pathway. CRL-2843-CD163 was transfected with 3×Flag-DAP12 and siNMHC-IIA or siRNA-NC for 48 h and infected by PRRSV (MOI = 5) for indicated time periods (0 and 1 h) (B). PAMs were infected by PRRSV (MOI = 1) for 1 h in the presence of blebbistatin (5 μM) (C). DAP12 was immunoprecipitated by anti-DAP12 MAb, and IB was performed with indicated antibodies. (D) *MYH9* knockdown assay, which provides supplemental data for Fig. 3B. PAMs with *MYH9* knockdown were infected with PRRSV (MOI = 0.1) for 1 h. qRT-PCR was performed to evaluate the knockdown. (E) Inhibition of NMHC-IIA activity promoted PRRSV-triggered NF-κB and MAPK activation. PAMs were infected with PRRSV (MOI = 1) in the presence of NMHC-IIA inhibitor blebbistatin (5 μM). IB was performed to detect indicated proteins in NF-κB and MAPK pathways. (F and G) DAP12 overexpression inhibited PRRSV-induced transcription of proinflammatory cytokines, which is restored by *MYH9* or *Syk* knockdown. CRL-2843-CD163 cells were transfected with 3×Flag-DAP12 (1 μg) and siNMHC-IIA or siSyk for 48 h and infected with PRRSV (MOI = 5) for 3 h. qRT-PCR was performed to detect mRNA abundance of TNF-α, IL-6, IL-8, and IL-1β (F). IB was used to determine the knockdown efficiencies (G). Experiments in all panels were repeated at least three times, and similar results were obtained. Quantitation data are mean ± SD (*n* = 3). Statistical analysis used in qRT-PCR was performed using the Student *t* test: *, *P* < 0.05; **, *P* < 0.01; ns, not significant. Download FIG S8, TIF file, 2.2 MB. Copyright © 2019 Liu et al. 2019 Liu et al. This content is distributed under the terms of the Creative Commons Attribution 4.0 International license.

In addition, noncytotoxic *MYH9* knockdown (Fig. S8D) augmented PRRSV-induced proinflammatory cytokine transcription (Fig. 3B). We also observed that NMHC-IIA inhibition (Fig. S8E) and *MYH9*, *DAP12*, or *Syk* knockdown (Fig. 3C) enhanced the activation of NF-κB, p38, and ERK1/2 triggered by PRRSV. Though DAP12 overexpression antagonized PRRSV-induced proinflammatory responses, knockdown of NMHC-IIA or Syk subverted the inhibitory function of DAP12 (Fig. 3D and E; Fig. S8F and G). Taken together, the NMHC-IIA-DAP12-Syk pathway was involved in repressing PRRSV-triggered proinflammatory responses.

### NMHC-IIA recognizes the sialic acids on PRRSV to inhibit proinflammatory responses via the DAP12-Syk pathway.

**FIG 4:**
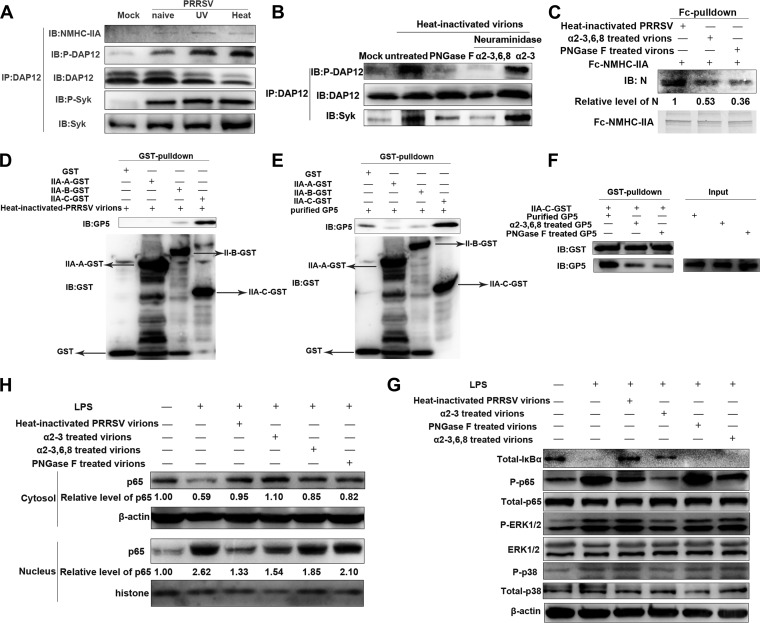
Sialic acids on PRRSV are recognized by the NMHC-IIA–DAP12-Syk pathway to suppress PRRSV-triggered proinflammatory responses. (A) Neither PRRSV structural glycoproteins nor viral genome induces activation of the DAP12-Syk pathway. PAMs were inoculated with the same amounts (MOI = 5) of naive, UV-inactivated, and heat-inactivated PRRSV virions for 1 h. IB was performed to detect the abundance of NMHC-IIA, DAP12, and Syk, as well as phosphorylated DAP12 and Syk. (B) Heat-inactivated PRRSV virions induce DAP12-Syk activation dependent on sialic acids. The virions were treated with PNGase F, α2-3,6,8 neuraminidase or α2-3 neuraminidase S for 90 min and then were used to inoculate PAMs for 1 h. IB was used to detect phosphorylated DAP12 as well as total DAP12 and Syk. (C) NMHC-IIA interacts with PRRSV partially dependent on the sialic acids. Fc-NMHC-IIA in Fc pulldown was conducted with the same amounts of PNGase F-, α2-3,6,8 neuraminidase-, or α2-3 neuraminidase S-treated virions. IB was performed to detect PRRSV N. (D and E) IIA-C is responsible for the interaction of NMHC-IIA with PRRSV GP5. GST-tagged IIA-A, IIA-B, or II-C in GST pulldown was performed with PRRSV virions (D) or GP5 (E). Eluted proteins were subjected to IB. (F) The interaction of IIA-C and GP5 is partially dependent on the sialic acids. IIA-C–GST in GST pulldown was conducted with PNGase F- or α2-3,6,8 neuraminidase-treated GP5 followed by IB analysis. (G and H) Heat-inactivated virions antagonize LPS-triggered NF-κB activation dependent on their sialic acids. PAMs were stimulated with LPS (10 μg/ml) for 30 min in the presence of PNGase F-, α2-3,6,8 neuraminidase-, or α2-3 neuraminidase S-treated virions. IB was performed to detect the indicated proteins in NF-κB and MAPK pathways (G) and determine nuclear transportation of p65 (H). Experiments in all panels were repeated at least three times, and similar results were obtained.

To identify the ligands required for NMHC-IIA–DAP12-Syk pathway activation, we inoculated PAMs with the same amounts of naive, UV-inactivated, and heat-inactivated PRRSV virions, respectively. Almost identical to naive ones, both UV- and heat-inactivated virions induced phosphorylation of DAP12 and Syk, as well as the interaction of NMHC-IIA or Syk with DAP12 (Fig. 4A). Various amounts of heat-inactivated virions all enhanced DAP12 phosphorylation and Syk binding to DAP12 (Fig. S9A). *MYH9* knockdown significantly inhibited phosphorylation of DAP12 and Syk induced by heat-inactivated PRRSV (Fig. S9B). The results suggested that neither PRRSV structural proteins nor viral RNA genome affected activation of the DAP12-Syk pathway.

10.1128/mBio.00574-19.9 FIG S9 Sialic acids on PRRSV are recognized by the NMHC-IIA–DAP12-Syk pathway to suppress virus-triggered proinflammatory responses. (A and B) Heat-inactivated virions activate the NMHC-IIA-DAP12-Syk pathway. PAMs were inoculated with increasing amounts (MOI = 1, 5, 10, and 20) of virions for 1 h (A), or *DAP12*, *Syk*, or *MYH9* knockdown PAMs were treated with virions at an MOI of 5 for 1 h (B). DAP12 was immunoprecipitated by anti-DAP12 MAb. IB was performed to detect phosphorylated DAP12 as well as total NMHC-IIA and DAP12. (C) Mixtures of 3′-sialyllactose and 6′-sialyllactose compete against PRRSV to interact with NMHC-IIA. A competitive Fc pulldown assay was performed to evaluate the binding of sialic acid mimics to Fc-NMHC-IIA. (D) Heat-inactivated virions inhibit LPS-induced proinflammatory cytokine transcription depending on their sialic acids. PAMs were stimulated with LPS (10 μg/ml) for 2 h and then inoculated with differently treated virions for 1 h. Proinflammatory cytokine transcription was analyzed by qRT-PCR. (E) Sialic acid mimics inhibit proinflammatory cytokine transcription in *trans*. In α2-3,6,8 neuraminidase-treated PAMs, qRT-PCR was used to determine the inhibitory effects exerted by sialic acid mimics on PRRSV-induced *TNFA* and *IL1B* transcription. Experiments in all panels were repeated at least three times, and similar results were obtained. Quantitation data are mean ± SD (*n* = 3). Statistical analysis used in qRT-PCR was performed using the Student *t* test: *, *P* < 0.05; **, *P* < 0.01; ***, *P* < 0.001; ns, not significant. Download FIG S9, TIF file, 2.4 MB. Copyright © 2019 Liu et al. 2019 Liu et al. This content is distributed under the terms of the Creative Commons Attribution 4.0 International license.

We hypothesized that protein modifications (e.g., N-glycosylation) might play a role, as they are not influenced by heat or UV irradiation. Heat-inactivated PRRSV virions were first treated with peptide-*N*-glycosidase F (PNGase F) to remove all N-glycans (20). Inoculation of virions without N-glycans did not induce activation of the DAP12-Syk pathway in PAMs (Fig. 4B). As α2-3- and α2-6-linked sialic acids are characteristic N-glycans on PRRSV virions, heat-inactivated virions were further treated with α2-3 neuraminidase S or α2-3,6,8 neuraminidase to remove α2-3- and α2-3,6-linked sialic acids, respectively (20). The virions where α2-6-linked sialic acids remained induced the activation of the DAP12-Syk pathway as the naive ones did, while the virions with removal of α2-3,6-linked sialic acids failed to activate the DAP12-Syk pathway (Fig. 4B). These results demonstrated that sialic acids on PRRSV were required for activation of the DAP12-Syk pathway.

To further elucidate whether sialic acids were the ligands of NMHC-IIA, we conducted Fc pulldown assays with Fc-fused NMHC-IIA and purified PRRSV virions treated with the indicated enzymes (21). The results indicated that PRRSV interacted with NMHC-IIA partially dependent on the α2-3,6-linked sialic acids, because the removal of N-glycans or α2-3,6-linked sialic acids decreased by about 50% or 60% the amount of PRRSV bound to NMHC-IIA, respectively (Fig. 4C). In addition, competitive Fc pulldown showed that the mixtures of sialic acid mimics (3′-sialyllactose or 6′-sialyllactose mimicking α2-3- or α2-6-linked sialic acids) competed against PRRSV to interact with NMHC-IIA (Fig. S9C). Moreover, NMHC-IIA nonhelical tail IIA-C was demonstrated to strongly interact with PRRSV in GST pulldown assays (Fig. 4D). PRRSV glycoprotein 5 (GP5) modified with sialic acids was shown to be important for PRRSV infection *in vitro* and *in vivo* (22, 23). We conducted pulldown assays with naive or PNGase F- or α2-3,6,8 neuraminidase-treated GP5 and IIA-C (21) and observed that GP5 interacted with IIA-C partially dependent on the sialic acids (Fig. 4E and F). The above results identified that the sialic acids on PRRSV were the ligands of NMHC-IIA.

We also evaluated the effects of heat-inactivated PRRSV virions on LPS-stimulated proinflammatory responses. The virions attenuated NF-κB activation (Fig. 4G and H) as well as *TNFA*, *IL-6*, and *IL1B* transcription in response to LPS (Fig. S9D). In contrast, virions with removal of N-glycans or α2-3,6-linked sialic acids lost the inhibitory function (Fig. 4G and H and Fig. S9D). Considering that sialic acids on the cell surface might *cis-*interact with NMHC-IIA, we removed sialic acids on PAMs with α2-3,6,8 neuraminidase treatment before LPS stimulation. Sialic acid mimics still inhibit LPS-induced transcription of proinflammatory cytokines in *trans* (Fig. S9E).

Taken together, these results showed that NMHC-IIA recognized sialic acids on PRRSV to repress the proinflammatory responses via the DAP12-Syk pathway.

### NMHC-IIA recognizes the sialic acids on VSV to inhibit proinflammatory responses by activating the DAP12-Syk pathway.

**FIG 5:**
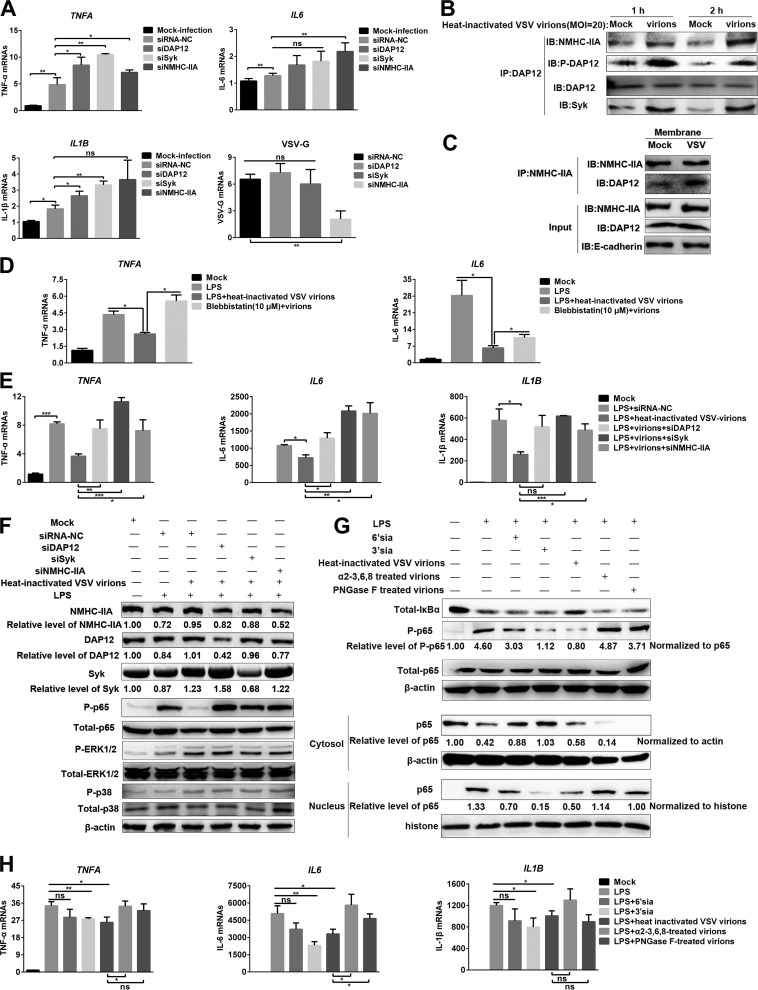
Sialic acids on VSV are recognized by NMHC-IIA to activate the DAP12-Syk pathway for inhibition of inflammatory responses. (A) The NMHC-IIA–DAP12-Syk pathway is involved in VSV-triggered proinflammatory cytokine transcription. RAW 264.7 cells with *MYH9*, *DAP12*, or *Syk* knockdown were infected with VSV (MOI = 1) for 1 h. qRT-PCR was performed to measure mRNA abundance of TNF-α, IL-6, IL-1β, and VSV-G. (B) Heat-inactivated VSV virions induce activation of the DAP12-Syk pathway. RAW 264.7 cells were inoculated with heat-inactivated virions (MOI = 20) for indicated time periods (1 and 2 h). DAP12 was immunoprecipitated by anti-DAP12 MAb. IB was performed to detect phosphorylated DAP12, as well as abundance of NMHC-IIA, DAP12, and Syk. (C) Heat-inactivated VSV virions increase association of NMHC-IIA and DAP12 on the membranes. RAW 264.7 cells were treated with heat-inactivated virions (MOI = 20) for 1 h. Membrane proteins were extracted to conduct an IP assay with anti-NMHC-IIA MAb. IB was then performed to detect the indicated proteins. (D) NMHC-IIA inhibitor subverted heat-inactivated VSV-mediated inhibition of proinflammatory cytokine transcription. LPS-stimulated RAW 264.7 cells were inoculated with the virions (MOI = 20) for 1 h in the presence of 5 μM blebbistatin and then subjected to qRT-PCR detection. (E and F) Heat-inactivated VSV virions inhibit LPS-induced NF-κB activation through the NMHC-IIA–DAP12-Syk pathway. RAW 264.7 cells with *MYH9*, *DAP12*, or *Syk* knockdown were stimulated with LPS (10 μg/ml) for 1 h and then treated with heat-inactivated VSV virions (MOI = 20) for 1 h. qRT-PCR analysis was applied to measure the mRNA abundance of TNF-α, IL-6, and IL-1β (E). IB was used to examine the indicated proteins in NF-κB, p38, and ERK1/2 pathways (F). (G and H) Heat-inactivated VSV virions suppress LPS-induced proinflammatory responses dependent on sialic acids. LPS (10 μg/ml)-stimulated RAW 264.7 cells were inoculated with PNGase F- or α2-3,6,8 neuraminidase-treated virions (MOI = 20) for 1 h. IB was performed to detect NF-κB pathway proteins (G). qRT-PCR was performed to detect *TNFA*, *IL-6*, and *IL1B* transcription (H). Experiments in all panels were repeated at least three times, and similar results were obtained. Quantitation shown was from three independent experiments (mean ± SD from three replicates). Statistical analysis used in qRT-PCR was performed using Student’s *t* test: *, *P* < 0.05; **, *P* < 0.01; ***, *P* < 0.001; ns, not significant.

To determine whether other sialylated RNA viruses were recognized by the NMHC-IIA–DAP12-Syk pathway for suppressing proinflammatory responses, we used vesicular stomatitis virus (VSV) with abundant α2-3-linked sialic acids (24) to inoculate murine peritoneal macrophage-like RAW 264.7 cells. Noncytotoxic knockdown of NMHC-IIA, DAP12, or Syk enhanced the *TNFA* and *IL1B* transcription in response to VSV (Fig. 5A). However, knockdown of NMHC-IIA decreased the amounts of VSV virions after 1 h of incubation at 37°C (Fig. 5A), suggesting that NMHC-IIA might be involved in VSV invasion. Therefore, we used heat-inactivated VSV virions instead of naive ones in the following experiments. IP analysis showed that heat-inactivated VSV (MOI = 20) enhanced phosphorylation of DAP12 and binding of NMHC-IIA or Syk to DAP12 at indicated time points (Fig. 5B). The virions also augmented the association of NMHC-IIA with DAP12 on cell membranes (Fig. 5C). Next, we investigated their effects on LPS-triggered proinflammatory responses. The virions inhibited the LPS-triggered proinflammatory responses in control RAW 264.7 cells but not in NMHC-IIA inhibitor-treated or *NMHC-IIA*, *DAP12*, or *Syk* knockdown cells (Fig. 5D to F). Differently, MAPK activation was not influenced (Fig. 5F), and *IL-8* transcription was not detected (data not shown). Intriguingly, we found that sialic acid mimics exerted a similar function as the virions in response to LPS, and 3′-sialyllactose had a stronger inhibition of NF-κB activation and *IL-6* transcription than 6′-sialyllactose (Fig. 5G and H). Furthermore, PNGase F- or α2-3,6,8 neuraminidase-treated virions lost the inhibitory function (Fig. 5G and H). All these findings demonstrated that NMHC-IIA recognized sialic acids on VSV to suppress the proinflammatory responses via the DAP12-Syk pathway.

### Sialic acid mimics inhibit LPS-induced proinflammatory responses through the NMHC-IIA–DAP12-Syk pathway.

**FIG 6:**
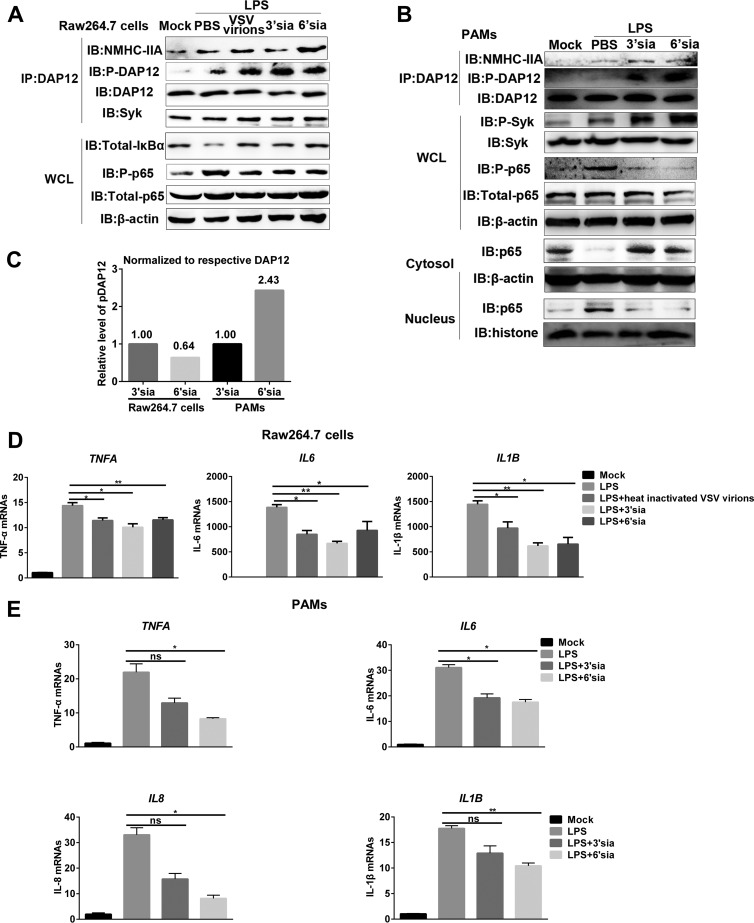
Sialic acid mimics activate NMHC-IIA–DAP12-Syk pathway to inhibit LPS-induced-inflammatory responses. (A to C) Sialic acid mimics activate NMHC-IIA–DAP12-Syk pathway and inhibit LPS-triggered NF-κB activation. LPS (10 μg/ml)-stimulated RAW 264.7 cells (A) or PAMs (B) were inoculated with 10 μM 3′-sialyllactose, 6′-sialyllactose, or heat-inactivated VSV virions (MOI = 20) for 1 h. An IP assay with anti-DAP12 MAb was conducted. IB analysis was performed to detect indicated proteins in NF-κB and NMHC-IIA–DAP12-Syk pathways. pDAP12 was normalized to immunoprecipitated DAP12, and the relative level of pDAP12 was analyzed by Image J (C). (D and E) Sialic acid mimics inhibit LPS-induced proinflammatory cytokine transcription. LPS (10 μg/ml)-stimulated RAW 264.7 cells were inoculated with 10 μM 3′-sialyllactose, 6′-sialyllactose, or heat-inactivated VSV (MOI = 20) for 1 h (D). LPS (10 μg/ml)-stimulated PAMs were inoculated with 10 μM 3′-sialyllactose or 6′-sialyllactose for 1 h (E). qRT-PCR was performed to detect transcription of TNF-α, IL-6, IL-8, and IL-1β. Experiments in all panels were repeated at least three times, and similar results were obtained. Quantitation data are mean ± SD (*n *= 3). Statistical analysis used in qRT-PCR was performed using Student’s *t* test: *, *P* < 0.05; **, *P* < 0.01; ns, not significant.

**FIG 7:**
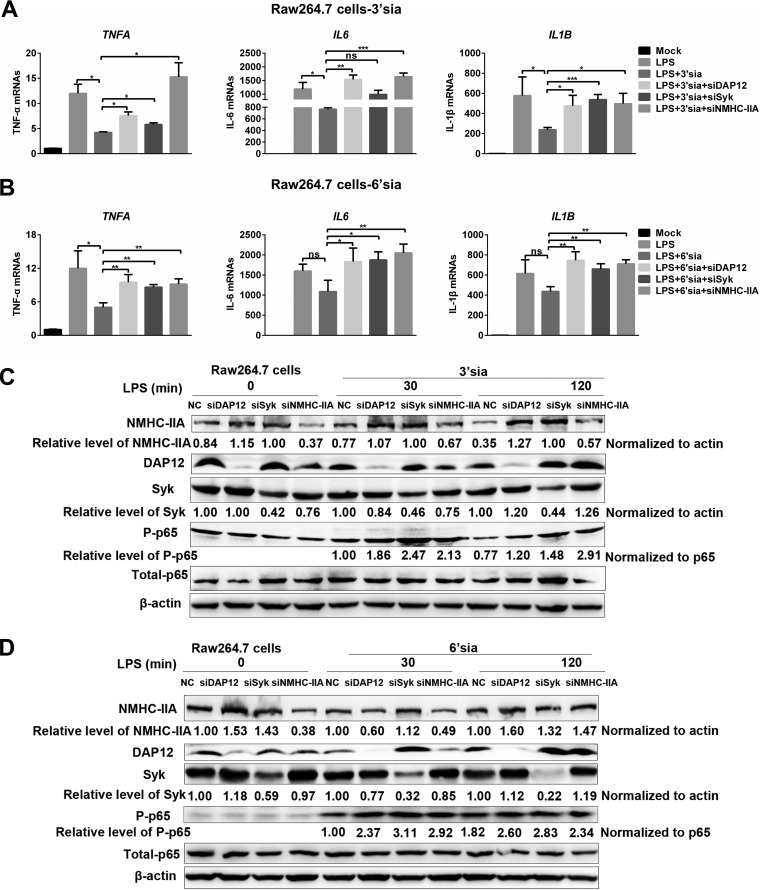
Sialic acid mimics (3′-sialyllactose and 6′-sialyllactose) inhibit LPS-induced inflammatory responses through the NMHC-IIA–DAP12-Syk pathway in RAW 264.7 cells. (A to D) RAW 264.7 cells were transfected with indicated siRNAs and stimulated with LPS (10 μg/ml) for different time periods (0, 30, and 120 min) in the presence of 3′-sialyllactose or 6′-sialyllactose (10 μM). IB was performed to detect NF-κB activation. Experiments in all panels were repeated at least three times, and similar results were obtained. Quantitation data are mean ± SD (*n* = 3). Statistical analysis used in qRT-PCR was performed using Student’s *t* test: *, *P* < 0.05; **, *P* < 0.01; ***, *P* < 0.001; ns, not significant.

**FIG 8:**
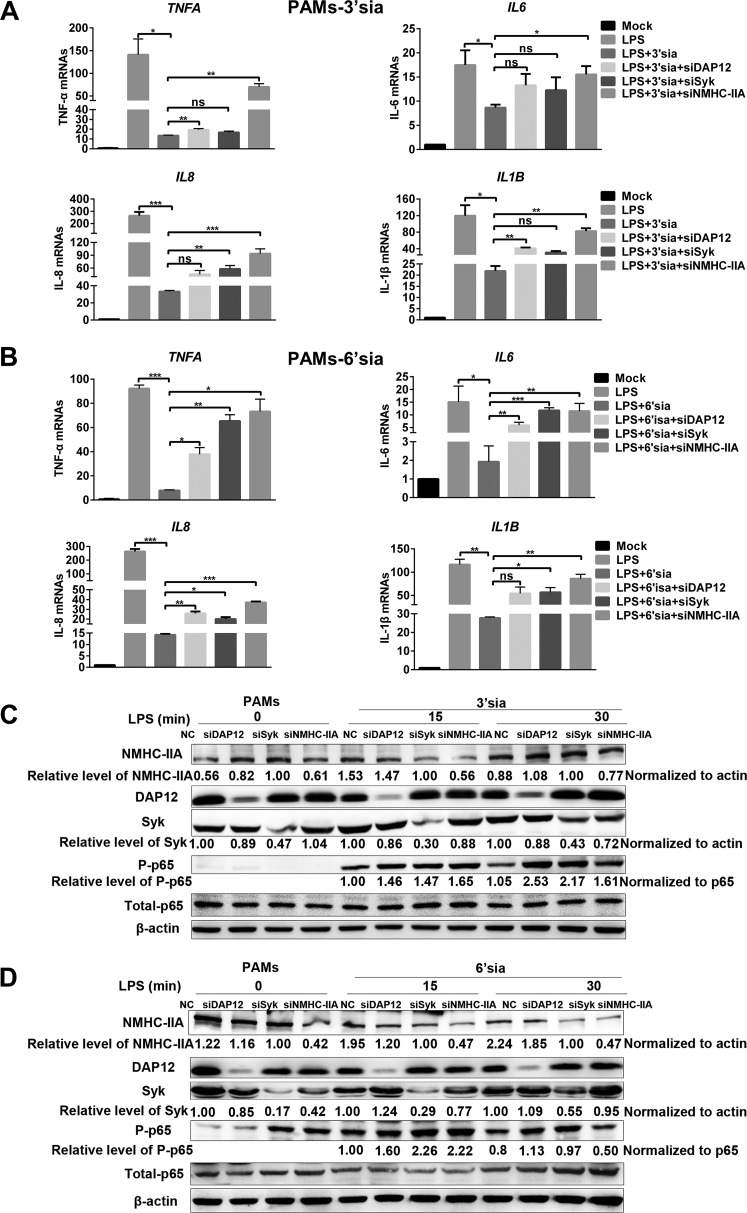
3′-Sialyllactose and 6′-sialyllactose both inhibit LPS-induced inflammatory responses via the NMHC-IIA–DAP12-Syk pathway in PAMs. (A to D) PAMs were transfected with indicated siRNAs targeting the NMHC-IIA–DAP12-Syk pathway and stimulated with LPS (10 μg/ml) for different time periods (0, 15, and 30 min) in the presence of 3′-sialyllactose or 6′-sialyllactose (10 μM). IB was performed to detect p65 phosphorylation. Experiments in all panels were repeated at least three times, and similar results were obtained. Quantitation data are mean ± SD (*n* = 3). Statistical analysis used in qRT-PCR was performed using Student’s *t* test: *, *P* < 0.05; **, *P* < 0.01; ***, *P* < 0.001; ns, not significant.

Based on the above results, we wondered whether sialic acids repressed LPS-induced proinflammatory responses through the NMHC-IIA–DAP12-Syk pathway independent of sialylated RNA viruses. Sialic acid mimics were shown to induce the activation of the NMHC-IIA–DAP12-Syk pathway and antagonize NF-κB activation (Fig. 6A and B). Interestingly, after analyzing the relative level of phosphorylated DAP12 (pDAP12) induced by sialic acid mimics, we found that pDAP12 levels in 3′-sialyllactose-treated RAW 264.7 cells were about twice those of 6′-sialyllactose-treated ones. In contrast, pDAP12 levels elevated by 6′-sialyllactose were about 2.4-fold higher than those elevated by 3′-sialyllactose in PAMs (Fig. 6C). We also observed that sialic acid mimics inhibited LPS-induced transcription of proinflammatory cytokines, and the inhibitory function of 3′-sialyllactose was stronger than 6′-sialyllactose in RAW 264.7 cells, while 6′-sialyllactose was the stronger one in PAMs (Fig. 6D and E). Additionally, sialic acid mimics suppressed LPS-triggered proinflammatory responses in RAW 264.7 cells (Fig. 7A to D) or PAMs (Fig. 8A to D). As expected, *MYH9*, *DAP12*, and *Syk* knockdown all terminated the inhibitory function of sialic acid mimics (Fig. 7 and 8) at different time points. Collectively, these results demonstrated that NMHC-IIA recognized sialic acids to inhibit LPS-induced proinflammatory responses by activating the NMHC-IIA–DAP12-Syk pathway.

## DISCUSSION

To eradicate invading viruses, various host signaling cascades are activated to induce effective proinflammatory responses. However, excessive host antiviral proinflammatory responses may develop into acute or chronic inflammatory disorders, and therefore, multiple negative regulatory mechanisms are needed to maintain homeostasis (25). In particular, Siglecs are a family of type I membrane proteins which specifically recognize sialic acid-modified glycans. It is known that several pathogens have evolved the capacity to gain sialic acids from their hosts and produce their own sialylated glycoconjugates (26). This capacity seems to be crucial for their survival in mammalian hosts, possibly by mimicking the host cell surface molecules to negatively regulate the innate immune responses and avoid the immune attack. For example, Siglec 1, Siglec H, and Siglec G were reported to be exploited by viruses to antagonize antiviral immune responses (27–29). Siglec 1 also played a role in establishing an immunosuppressive state of inflammation (30).

NMHC-IIA plays important roles in cell adhesion, cell migration, and cell division (31). Increasing evidence indicates that NMHC-IIA is required for entry of viruses such as herpes simplex virus 1 (13), Epstein-Barr virus (14, 32), severe fever with thrombocytopenia syndrome virus (33), and PRRSV (34). Here, we identified that NMHC-IIA functioned as a Siglec to negatively regulate NF-κB- and MAPK-mediated proinflammatory responses. GST pulldown assays demonstrated that NMHC-IIA directly interacted with sialylated RNA viruses partially dependent on sialic acids (Fig. 4; see also Fig. S9 in the supplemental material). Furthermore, knockdown of NMHC-IIA led to augmented transcription of proinflammatory cytokines and activation of NF-κB, p38, and ERK1/2 in response to sialylated RNA viruses (Fig. 3; Fig. S5 and S8). To distinguish the immunoregulation function from its role in viral invasion, we used heat-inactivated virions or monovalent sialic acid mimics instead of naive viruses to inoculate LPS-stimulated cells, which still resulted in decreased proinflammatory cytokine production and attenuated NF-κB activation (Fig. 4 and 8 and Fig. S9). In contrast, *MYH9* knockdown recovered the proinflammatory responses (Fig. 5 and 8). Interestingly, we found that the suppression of LPS-triggered inflammation by sialic acid mimics in RAW 264.7 cells was different from that in PAMs. 3′-Sialyllactose showed higher inhibitory potency than 6′-sialyllactose in RAW 264.7 cells, while 6′-sialyllactose was the more potent one in PAMs. The difference might be due to cell species. We did not obtain the appropriate multivalent sialic acid mimics to investigate their effects on LPS-triggered proinflammatory responses, which needed a further exploration. These findings revealed a novel role of NMHC-IIA in negative modulation of innate immune responses and may be applied to design of anti-inflammatory drugs.

DAP12, also called TYROBP (tyrosine kinase binding protein) and KARAP (killer cell activating receptor-associated protein), functions as an adaptor in various immune cells, including macrophages, microglia, monocytes, DCs, and natural killer (NK) cells (35). DAP12 contains a small ECD, a TMD, and an ICD. The ECD possesses an essential cysteine required for the homodimer formation, the TMD possesses an aspartic acid indispensable for interaction with DAP12-associated receptors, and the ICD possesses an ITAM responsible for interaction with Syk and delivering signals (18). The receptor-DAP12-Syk axis is a classic pathway involved in immune responses such as synergistic activation of proinflammatory cytokine production (36) and IFN augmentation (37). Interestingly, the axis is also involved in negative regulation of proinflammatory responses. *DAP12*- and *Syk*-deficient macrophages from the corresponding knockout mice displayed an increased secretion of proinflammatory cytokines in response to LPS, CpG DNA, and synthetic lipopeptide (38). In our study, we observed activation of the DAP12-Syk pathway during viral early infection (Fig. 1 and 5). Knockdown of DAP12 or Syk enhanced both virus-triggered production of proinflammatory cytokines and NF-κB/MAPK activation (Fig. 1 and 5; Fig. S1 and S4). In contrast, DAP12 overexpression antagonized these responses (Fig. S2). These results suggested that the DAP12-Syk pathway was hijacked to suppress the antiviral proinflammatory responses.

So far, only the triggering receptor expressed on myeloid cells 2 (TREM-2) was identified to be a DAP12-associated receptor mediating inhibition of Toll-like receptor (TLR) and FcR signaling (39). DAP12-associated receptors in negative regulation of proinflammatory responses were not fully illustrated. Here, we identified a new DAP12-associated receptor, NMHC-IIA, through IP-MS, IFA, and co-IP assays (Fig. 2 and Fig. S6). The results showed a direct interaction between NMHC-IIA and DAP12, with a requirement of NMHC-IIA domain B (mainly containing α-helices) and a short stretch (residues 51 to 57) within the DAP12 TMD (Fig. 2 and Fig. S7). In addition, we identified the sialic acids on RNA viruses (VSV and PRRSV) as the ligands of NMHC-IIA to activate the DAP12-Syk pathway (Fig. 4 and 5 and Fig. S9).

**FIG 9:**
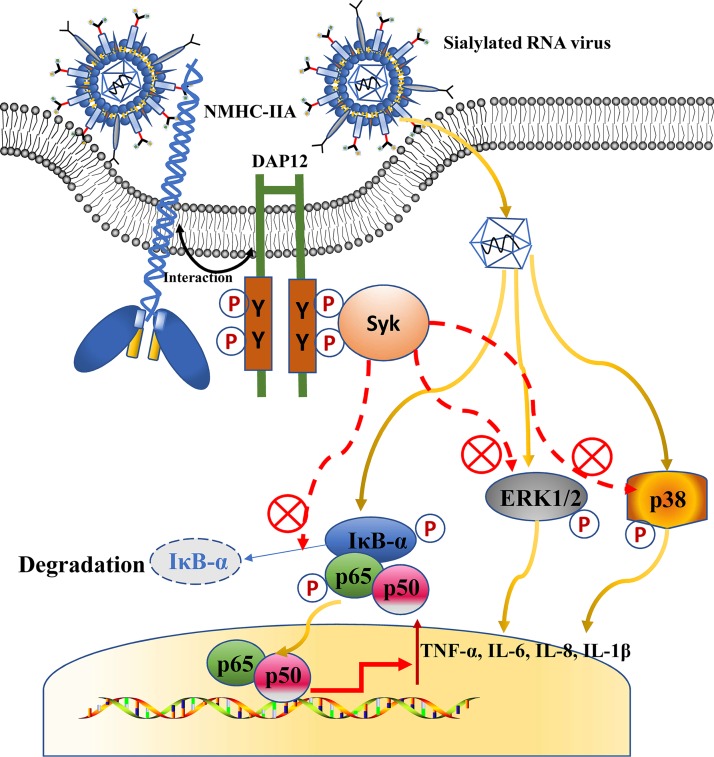
Sialic acids on sialylated RNA viruses are recognized by NMHC-IIA to suppress antiviral proinflammatory responses. Upon infection, NMHC-IIA recognizes and interacts with sialylated RNA viruses to activate the DAP12-Syk pathway. Activation of the NMHC-IIA–DAP12-Syk pathway subsequently antagonizes NF-κB- and MAPK-mediated proinflammatory responses.

PRRSV, a sialylated RNA virus, has been shown to inhibit NF-κB-mediated inflammatory responses during early infection while triggering the responses in the late infection (40). VSV, another sialylated RNA virus (41), has also been reported to induce a delayed NF-κB activation (42). Consequently, the two sialylated RNA viruses are utilized to explore the interaction between sialic acids and host innate immunity during viral infections. Knockdown of NMHC-IIA, DAP12, and Syk all promoted virus-triggered proinflammatory responses (Fig. 1 and 3 and Fig. S8). We also found that *DAP12* knockdown promotes proinflammatory cytokine transcription at time zero (Fig. 1), which might be explained by DAP12 being involved in negative regulation of immune responses for maintaining homeostasis (16, 43). However, *IL-10* transcription was not induced during PRRSV infection, a finding which was different from previous reports where PRRSV upregulated IL-10 expression (44, 45). This divergence might be due to the PRRSV strains and PAMs used and needs to be further elucidated. Next, we identified that sialic acids on viruses were the stimuli and exerted the anti-inflammatory effects (Fig. 4 and 5 and Fig. S9). This may be a common mechanism in negative regulation of antiviral proinflammatory responses (Fig. 9), which needs further demonstration in other sialylated RNA viruses, and even DNA viruses or bacteria.

Indeed, there are some issues left unsolved in the current work. First, we performed knockdown instead of knockout of NMHC-IIA *in vitro* since *MYH9* knockdown with more than 70% efficiency was detrimental to the cells (Fig. S8). We failed to get the *MYH9* knockout mice and perform *in vivo* experiments because the loss of NMHC-IIA was lethal for mice, which is consistent with a previous study (46). Second, it might be better to generate a stable cell line with *DAP12* or *Syk* knockdown to determine their inhibitory functions. However, it is hard to obtain such stable cell lines for primary PAMs. Third, factors downstream of Syk delivering the inhibitory signals have not been discovered, and the related work is being carried out in our laboratory. Fourth, DAP10 is similar to DAP12 in its TMDs. There are certain receptors associated with DAP12 which are also identified to pair with DAP10 (43). Whether DAP10 is associated with NMHC-IIA needs to be further studied. Our initial work showed that *DAP12* knockdown increased type I IFN production. Whether NMHC-IIA–DAP12-Syk has effects on IFN responses is our next issue to be explored.

In conclusion, we identify that NMHC-IIA recognizes sialic acids on sialylated RNA viruses and activates the DAP12-Syk pathway to suppress virus-triggered proinflammatory responses. More importantly, the NMHC-IIA–DAP12-Syk pathway is shown to inhibit LPS-induced proinflammatory responses on recognition of heat-inactivated sialylated RNA viruses and sialic acid mimics. Taken together, we have revealed a novel negative regulation mechanism of proinflammatory responses, which expands our knowledge of the host innate immune system and provides clues for struggling with the sialylated RNA viruses and inflammatory diseases.

## MATERIALS AND METHODS

### Ethics statement.

All experiments were performed according to the Chinese Regulations of Laboratory Animals—*The Guidelines for the Care of Laboratory Animals* (Ministry of Science and Technology of People’s Republic of China). The experimental procedure for collection of PAMs was authorized and supervised by the Ethical and Animal Welfare Committee of Key Laboratory of Animal Immunology of the Ministry of Agriculture of China (permit no. 2016001).

### Cells and viruses.

PAMs were collected from lung lavage samples of 4-week-old specific-pathogen-free (SPF) pigs. CRL-2843 (3D4/21), MARC-145 (a monkey kidney cell line subcloned from MA-104), HEK-293T, and RAW 264.7 cell lines were purchased from Cellbio (Shanghai, China). CRL-2843-CD163, a cell line stably expressing CD163 in CRL-2843, was constructed in our laboratory. PAMs and CRL-2843-CD163 cells were routinely maintained in Roswell Park Memorial Institute 1640 medium (RPMI 1640) supplemented with 10% heat-inactivated fetal bovine serum (FBS; Gibco), penicillin (100 U/ml), and streptomycin (100 mg/ml) in a humidified 37°C, 5% CO_2_ incubator. MARC-145, HEK-293T, and RAW 264.7 cells were maintained in Dulbecco’s modified Eagle’s medium (DMEM) supplemented with 10% FBS and antibiotics.

Highly pathogenic (HP) PRRSV strain HN07-1 (isolated during an HP-PRRSV outbreak in the Henan province of China in 2007 by our laboratory) and VSV (Indiana serotype kept in our laboratory) were prepared according to our laboratory’s previous study (47). UV-inactivated viruses were generated by irradiation with short-wave UV light (254 nm) for 1 h, and heat-inactivated ones were gained by a water bath at 65°C for 15 min. The viral infectivity was confirmed to be completely lost. The viruses were purified through tangential flow filtration and Sepharose 4 Fast Flow gel chromatography, and their infectivity was comparable to that of naive ones.

### Antibodies and reagents.

The antibodies used were as follows: Myc-tag (9B11) mouse monoclonal antibody (MAb) (catalog no. 2276), Myc-tag (71D10) rabbit MAb (catalog no. 2278), Flag (DYKDDDDK)-tag (D6W5B) rabbit MAb (catalog no. 14793), Flag (DYKDDDDK) tag (9A3) (catalog no. 8146), β-actin (8H10D10) mouse MAb (catalog no. 3700), NF-κB p65 (D14E12) XP rabbit MAb (catalog no. 8242), phospho-NF-κB p65 (Ser536) (93H1) rabbit MAb (catalog no. 3033), IκBα (L35A5) mouse MAb (amino-terminal antigen) (catalog no. 4814), phospho-IκBα (Ser32/36) (5A5) mouse MAb (catalog no. 9246), Syk (D3Z1E) XP rabbit MAb (catalog no. 13198), phospho-Syk (Tyr525/526) (C87C1) rabbit MAb (catalog no. 2710), p44/42 MAPK (ERK1/2) (137F5) rabbit MAb (catalog no. 4695), phospho-p44/42 MAPK (ERK1/2) (Thr202/Tyr204) (D13.14.4E) XP rabbit MAb (catalog no. 4370), p38 MAPK antibody (catalog no. 9212), phospho-p38 MAPK (Thr180/Tyr182) antibody (catalog no. 9211), DAP12 (D7G1X) rabbit MAb (catalog no. 12492), and E-cadherin (24E10) rabbit MAb (catalog no. 3195) were all purchased from Cell Signaling Technology (Danvers, MA, USA). DAP12 antibody (G-5) (catalog no. sc-133174) and Syk antibody (D-3) (catalog no. sc-166226) were purchased from Santa Cruz Biotechnology (Santa Cruz, CA, USA). GST tag mouse MAb (catalog no. 66001-2-Ig) and histone-H3 rabbit polyclonal antibody (catalog no. 17168-1-AP) were all purchased from Proteintech (Wuhan Sanying, China). Anti-human IgG antibody (EPR4421) (catalog no. ab109489) was purchased from Abcam (Cambridge, United Kingdom). Antiphosphotyrosine antibody, clone 4G10 (catalog no. 05-321), was purchased from Merck Millipore (Ontario, Canada). Monoclonal anti-MYH9 antibody produced in mouse (catalog no. SAB1404102) was purchased from Sigma-Aldrich (St. Louis, MO, USA). Purified mouse anti-E-cadherin clone 36 (catalog no. 610182) was purchased from BD Biosciences (Gurgaon, Haryana, India). Mouse MAbs against PRRSV nucleocapsid (N) and glycoprotein (GP) 5 were kept in our laboratory.

Reagents used were as follows: protein A Sepharose 4 Fast Flow (catalog no. 17-5280-01; GE Healthcare, Pittsburgh, PA, USA), protein G Sepharose 4 Fast Flow (catalog no. 17-0618-01; GE Healthcare), DyLight 350 goat anti-mouse IgG (catalog no. A23010; Abbkine, Wuhan, China), ERK inhibitor PD 98059 (catalog no. 513000; Calbiochem, San Diego, CA, USA), JNK inhibitor II-129566 (catalog no. 420119; Calbiochem), p38 MAP kinase inhibitor SB253080 (catalog no. tlrl-sb20; InvivoGen, Hong Kong, China), NF-κB inhibitor BAY11-7082 (catalog no. tlrl-b82; Beyotime Biotechnology, Shanghai, China), 3′-sialyllactose (catalog no. A8681; Sigma-Aldrich), 6′-sialyllactose sodium salt (catalog no. A8556; Sigma-Aldrich), lipopolysaccharides (LPS) from Escherichia coli O111:B4 (catalog no. L3024; Sigma-Aldrich), (±)-blebbistatin (catalog no. 760; Tocris Bioscience, Bristol, United Kingdom), α2-3,6,8 neuraminidase (catalog no. P0720; New England Biolabs, Ipswich, MA), α2-3 neuraminidase S (catalog no. P0743; New England Biolabs), PNGase F (catalog no. P0704; New England Biolabs), Syk inhibitor R406 (catalog no. S2194; Selleck, Houston, TX, USA), Pierce protease and phosphatase inhibitor minitablets (catalog no. A32959; Thermo Fisher Scientific, Waltham, MA, USA), PrimeScript reverse transcription (RT) reagent kit with genomic DNA (gDNA) Eraser (catalog no. RR047B; TaKaRa, Dalian, China), FastStart Universal SYBR Green Master (Rox) (catalog no. 4913850001; Roche, Basel, Switzerland), porcine TNF-α Quantikine enzyme-linked immunosorbent assay (ELISA) kit (catalog no. PTA00; R&D Systems), CellTiter 96 AQueous One Solution cell proliferation assay (MTS) (catalog no. G3582; Promega, Madison, WI, USA), nuclear and cytoplasmic protein extraction kit (catalog no. P0028; Beyotime), ProteoExtract native membrane protein extraction kit (catalog no. 444810; Merck Millipore).

### RNA interference.

All small interference RNAs (siRNAs) and siRNA-negative control (NC) were designed and synthesized by GenePharma (Shanghai, China). In knockdown experiments, PAMs or CRL-2843-CD163 or RAW 264.7 cells were transfected with the indicated siRNAs at a final concentration of 10 nM or 20 nM using Lipofectamine RNAiMAX according to the manufacturer’s instructions (Invitrogen) for 36 h or 48 h. Transfected cells were then applied for subsequent experiments after cell viability measurement by the CellTiter 96 AQueous One Solution assay according to the manufacturer’s instructions. The indicated siRNAs are listed in Table 1.

**TABLE 1:** siRNAs in this study

Target gene	5′–3′ (sense)	5′–3′ (antisense)
Pig DAP12-433	GGAUACGGAUCCACAGAGUTT	ACUCUGUGGAUCCGUAUCCTT
Pig Syk-443	GGUAGCGUAUGACAGGAAGTT	CUUCCUGUCAUACGCUACCTT
Pig MYH9-249	GCAAGCCGCCGAUAAGUAUTT	AUACUUAUCGGCGGCUUGCTT
Mouse DAP12-148	CCAAGAUGCGACUGUUCUUTT	AAGAACAGUCGCAUCUUGGTT
Mouse Syk-407	CCAUCGAGAGGGAACUUAATT	UUAAGUUCCCUCUCGAUGGTT
Mouse MYH9-591	GUGGUCAUCAACCCUUAUATT	UAUAAGGGUUGAUGACCACTT
siRNA-NC	UUCUCCGAACGUGUCACGUTT	ACGUGACACGUUCGGAGAATT

### qRT-PCR.

Total RNAs were extracted with TRIzol reagent (Invitrogen), and the reverse transcription cDNAs were prepared from total RNAs using the PrimeScript RT reagent kit with gDNA Eraser (TaKaRa). The cDNAs from different samples were ampliﬁed by quantitative real-time PCR (qRT-PCR) to measure mRNA abundance on a 7500 Fast RT-PCR system (Applied Biosystems, Foster City, CA, USA). Relative mRNA level was evaluated by the 2^−ΔΔ^*^CT^* method with glyceraldehyde-3-phosphate dehydrogenase (GAPDH) mRNA as an endogenous control (48). The primers for qRT-PCR analysis are listed in Table S1 in the supplemental material.

10.1128/mBio.00574-19.10 TABLE S1 Primers for qRT-PCR and expression vector construction in this study. Download Table S1, DOC file, 0.02 MB. Copyright © 2019 Liu et al. 2019 Liu et al. This content is distributed under the terms of the Creative Commons Attribution 4.0 International license.

### IB and IP.

Cells were harvested and lysed in radioimmunoprecipitation assay (RIPA) lysis buffer (Beyotime Biotechnology), supplemented with protease and phosphatase inhibitors. Whole-cell lysates (WCLs) were normalized to equal amounts of β-actin, separated by 8 to 15% gradient sodium dodecyl sulfate-polyacrylamide gel electrophoresis (SDS-PAGE), and electrotransferred onto Immobilon-P membranes (Merck Millipore). The membranes were blocked in 5% skimmed milk for 1 h and probed with the indicated primary antibodies. After incubation with horseradish peroxidase (HRP)-labeled goat anti-mouse or rabbit IgG antibody as secondary antibodies, the indicated proteins were visualized with enhanced chemiluminescence (ECL) reagent (Solarbio). For IP, the indicated primary antibodies were first bound to protein A/G beads at 4°C for 4 h. Samples were subsequently incubated with the beads at 4°C overnight, and potential associated proteins were tested by IB as stated above. The relative levels of target proteins were analyzed using Image J software, and the ratio was displayed as fold change below the images.

### ELISA.

PAMs with *DAP12* knockdown were inoculated with PRRSV at an MOI of 0.1 for indicated time periods (3, 6, 9, 12, 24, and 36 h). The cell supernatants were collected for measurement of TNF-α using ELISA kits according to the manufacturer’s instructions.

### Virus titration assay.

The transfected cells were inoculated with PRRSV at an MOI of 0.1, 1, or 5. At indicated time points (24, 36, and 48 h) postinfection, the progeny virus titers were measured by the 50% tissue culture infective dose (TCID_50_) assay in MARC-145 cells.

### Expression vector construction.

Complete NMHC-IIA cDNA was obtained from pTrip-MYH9 (34). NMHC-IIA was subcloned into the vector pcDNA3-EGFP (provided by Doug Golenbock; Addgene plasmid no. 13031) and pFUSE-hIgG1-Fc2 (InvivoGen) and named EGFP-NMHC-IIA and Fc-IIA, respectively. Three fragments of NMHC-IIA, IIA-A (residues 1 to 742), IIA-B (residues 743 to 1560), and IIA-C (residues 1561 to 1957), were subcloned into the vectors pFUSE-hIgG1-Fc2 and pGEX-6P-1 (GE Healthcare) and designated Fc-IIA-A, Fc-IIA-B, and Fc-IIA-C or GST-IIA-A, GST-IIA-B, and GST-IIA-C, respectively. The cDNAs encoding DAP12 and Syk were obtained from PAM cDNA and subcloned into pCMV7.1-3×Flag vector and pcDNA3.1-myc-hisA vector (Invitrogen), respectively. The cDNA encoding DAP12 was also inserted into pcDNA3-mRFP, which was provided by Doug Golenbock (Addgene plasmid no. 13032). The DAP12 mutants (DAP12-2Y-2F, DAP12-5Y-5F, and DAP12-D50A) were generated and inserted into the pCMV7.1-3×Flag vector. The two truncated DAP12s (DAP12-ΔICD and DAP12-ΔECD) and three fragments of DAP12 with partial deletion in the TMD (DAP12-ΔTM1, -ΔTM2, and -ΔTM3) were inserted into pCMV7.1-3×Flag vector. These constructs were designated Syk-myc-his, 3×Flag-DAP12, DAP12-mRFP, 3×Flag-DAP12 (D50A), 3×Flag-DAP12 (2Y-2F), 3×Flag-DAP12 (5Y-5F), 3×Flag-DAP12-ΔECD, 3×Flag-DAP12-ΔICD, 3×Flag-DAP12-ΔTM1, 3×Flag-DAP12-ΔTM2, and 3×Flag-DAP12-ΔTM3. Plasmid pCAGGS-GP5-Flag was kept in our laboratory. All constructs were verified by Sangon Biotech Co. Ltd. (Shanghai, China). The primers for expression vector construction are listed in Table S1.

### Protein expression and purification.

Prokaryotic expression was performed in E. coli BL21(DE3) cells (TransGen Biotech, Beijing, China). The expression of each target protein was induced with 0.5 mM isopropyl-β-d-thiogalactoside (IPTG; Solarbio) at 18°C overnight. The centrifuged cells were resuspended and sonicated in 20 mM Tris-HCl, pH 6.8, 150 mM NaCl, 2 mM MgCl_2_. The protein was purified by GST resin (GenScript Biotech Corp., Nanjing, China) and eluted by 50 mM Tris-HCl, pH 8.0, 10 mM reduced glutathione.

Eukaryotic expression was conducted by transfection of each expression vector into HEK-293T or CRL-2843-CD163 cells for 36 h using Lipofectamine 2000 or Lipofectamine LTX with Plus reagent according to the manufacturer’s instructions (Thermo Fisher Scientific). The transfected cells were lysed in RIPA lysis buffer supplemented with protease and phosphatase inhibitors and clarified by centrifugation at 12,000 rpm at 4°C for 15 min to collect supernatants. Protein A/G beads were incubated with the indicated antibodies and WCLs or cell supernatants at 4°C and eluted by 0.05 M glycine-HCl buffer, pH 2.2 (0.2 M glycine, 0.2 M HCl).

### Co-IP.

HEK-293T or CRL-2843-CD163 cells were lysed with 1 ml NP-40 lysis buffer (20 mM Tris-HCl, pH 7.4, 150 mM NaCl, 1 mM EDTA, 1 mM sodium orthovanadate, 1% Nonidet P-40, 10 mg/ml aprotinin, 10 mg/ml leupeptin, and 1 mM phenylmethylsulfonyl fluoride) for 30 min on ice. WCLs were centrifuged at 12,000 rpm at 4°C for 15 min, and the supernatants were harvested. For each IP, the supernatants (0.8 ml) were incubated with the indicated antibodies (0.5 μg) and protein A/G beads (30 μl) at 4°C for 3 h. The protein-bound beads were then collected and washed three times with Tris-buffered saline (TBS), pH 7.4.

### Inhibitor treatments.

PAMs were seeded onto 24-well plates and treated with specific inhibitors of MAPKs (ERK1/2, p38, and JNK) and NF-κB at different concentrations (5, 10, 15, 20, 25, and 30 μM), Syk inhibitor at 5 μM for 12 h, or NMHC-IIA inhibitor at 20 μM for 1 h. Cell viability measurement was performed as stated above. Then, PAMs were inoculated with PRRSV at an MOI of 0.1 for 1 h, and transcription of proinflammatory cytokines was tested by qRT-PCR. Phosphorylation of indicated proteins was detected by IB.

### Mass spectrometry analysis.

PAMs were inoculated with PRRSV for 1 h and lysed for IP with DAP12 primary antibody or isotype control IgG. DAP12-associated proteins were eluted and subjected to SDS-PAGE and silver staining. Discrepant bands with intensive signal compared with isotype control IgG were cut and digested, followed by analysis using matrix-assisted laser desorption ionization–time of flight mass spectrometry (MALDI-TOF MS) by Shanghai Sangon Biotech Co. Ltd.

### IFA.

CRL-2843-CD163 cells were grown on coverslips in 6-well plates at 30 to 50% confluence. For visualization of the distribution of NMHC-IIA, DAP12, or Syk, cells were transfected with EGFP–NMHC-IIA or/and DAP12-mRFP for 36 h and then mock infected or infected with PRRSV (MOI = 20) for 1 h. The cells were fixed with 4% paraformaldehyde for 15 min at room temperature and then blocked with 5% BSA-PBST for 30 min. Next, cells were incubated with anti-Syk MAb at 4°C for 2 h, followed by incubation with DyLight 350 (blue)-conjugated anti-mouse IgG for an additional 45 min. The localization of NMHC-IIA, DAP12, or Syk was observed under an inverted fluorescence and phase-contrast microscope (Carl Zeiss AG, Oberkochen, Germany). Images were taken at a ×400 magnification.

### *In vitro* pulldown assays.

GST resins or protein A/G beads were incubated with purified GST- or Fc-tagged proteins at 4°C for 2 h and then with the indicated purified Flag-tagged proteins or differently treated virions at 4°C for another 30 min or overnight. After extensive washing with TBS 4 times, proteins were eluted and subjected to IB with the indicated antibodies.

### Deglycosylation treatments.

Heat-inactivated virions were treated with PNGase F in PBS at 37°C for 90 min to remove all N-glycans (high-mannose and complex, sialic acid-containing glycans). To remove sialic acids, the virions were incubated with specific neuraminidases in PBS at 37°C for 90 min or only the neuraminidase buffer (0 unit of neuraminidase) as a negative control. PAMs or RAW 264.7 cells were stimulated with LPS (10 μg/ml) for indicated time periods (0, 15, and 30 min or 0, 30, and 120 min) in the presence of deglycosylation virions for 1 h. After extensive washing, cells were collected to conduct protein or mRNA extraction. The removal of sialic acids on the purified PRRSV GP5 was performed in parallel. The desialylated protein was further applied for pulldown assays. To remove sialic acids on the cells before infection, PAMs or RAW 264.7 cells were incubated with α2-3,6,8 neuraminidase at 37°C for 1 h and washed extensively to remove the enzyme (20, 49).

### Competition experiments with monovalent sialic acid mimics.

The protein A/G beads with Fc-tagged NMHC-IIA were incubated with the purified PRRSV virions in the presence or absence of mixtures of 20 μM 3′-sialyllactose and 6′-sialyllactose sodium salt at 4°C for 3 h. The virions were eluted and measured by IB.

### Protein extraction.

The nuclear and cytoplasmic proteins were separately extracted using a nuclear and cytoplasmic protein extraction kit and applied for IB detection. The membrane and cytoplasmic proteins were harvested using the ProteoExtract native membrane protein extraction kit.

### Treatment with virions and sialic acid mimics.

PAMs were incubated with the same amount (MOI = 10 or 20) of heat-inactivated virions, desialylatd virions, or 10 μM sialic acid mimics (soluble 3′-sialyllactose and 6′-sialyllactose sodium salt) for 1 h and later stimulated with LPS (10 μg/ml) for indicated time periods (0, 15, and 30 min). RAW 264.7 cells were stimulated with LPS (10 μg/ml) at indicated time points (30 and 120 min) in the presence of the specifically treated virions or the sialic acid mimics for 1 h. The indicated proteins and proinflammatory cytokines were analyzed by IB and qRT-PCR, respectively.

### Statistical analysis.

Three replicates were included in all experiments, and each experiment was independently repeated at least three times. The experimental data were presented as group mean and standard deviation (SD) and analyzed by the unpaired two-tailed Student *t* test with GraphPad (GraphPad Software, San Diego, CA, USA). Asterisks indicate statistical significance as follows: ns, not significant; *, *P* < 0.05; **, *P* < 0.01; ***, *P* < 0.001.
