# Supplementary material for: Nonmuscle Myosin Heavy Chain IIA Recognizes Sialic Acids on Sialylated RNA Viruses To Suppress Proinflammatory Responses via the DAP12-Syk Pathway
Source: mBio. 2019 May 7;10(3):e00574-19. doi: 10.1128/mBio.00574-19 (PMC6509187; doi:10.1128/mBio.00574-19)
Supplement: TABLE S1 [file mBio.00574-19-st001.doc]

**TABLE S1 Primers for qRT-PCR and expression vector construction in this study.**

| Name | Primer (5’-3’) |
| --- | --- |
| pig GAPDH F: | CCTTCCGTGTCCCTACTGCCAAC |
| pig GAPDH R: | GACGCCTGCTTCACCACCTTCT |
| pig TNF-α-F: | CCCCCAGAAGGAAGAGTTTC |
| pig TNF-α-R: | CGGGCTTATCTGAGGTTTGA |
| pig IL-6-F: | AATGTCGAGGCTGTGCAGATT |
| pig IL-6-R: | TGGTGGCTTTGTCTGGATTCT |
| pig IL-8-F: | GGCAGTTTTCCTGCTTTCT |
| pig IL-8-R: | CAGTGGGGTCCACTCTCAAT |
| pig IL-1β-F: | TCTGCCCTGTACCCCAACTG |
| pig-IL-1β-R: | CCCAGGAAGACGGGCTTT |
| pig-IL-10-F: | CGGCGCTGTCATCAATTTCTG |
| pig-IL-10-R: | CCCCTCTCTTGGAGCTTGCTA |
| pig DAP12-F | ACCCGGAAACAACACATCGC |
| pig DAP12-R | TACTGCCTCTGTGTGTTGAGG |
| pig Syk-F: | GGAAGGCGCATCACTACAC |
| pig Syk-R: | TGGCTGATAATTGCCTGCTCC |
| pig MYH9-F: | AAGGCACCGTCAAGTCCAA |
| pig MYH9-R: | TTCCTCCGCTCATCATCCA |
| VSV-G F: | ACGGCGTACTTCCAGATGG |
| VSV-G R; | CTCGGTTCAAGATCCAGGT |
| mouse GAPDH F: | AACTTTGGCATTGTGGAAGG |
| mouse GAPDH R: | ACACATTGGGGGTAGGAACA |
| mouse TNFα F | GCCACCACGCTCTTCTGTCT |
| mouse TNFα R | TGAGGGTCTGGGCCATAGAAC |
| mouse IL-6-F: | ACAACCACGGCCTTCCCTAC |
| mouse IL-6-R: | CATTTCCACGATTTCCCAGA |
| mouse-IL-8-F： | CCGTCCCTGTGACACTCAAG |
| mouse-IL-8-R： | ACAGAAGCTTCATTGCCGGT |
| mouse IL-1β-F: | GAAATGCCACCTTTTGACAGTG |
| mouse IL-1β-R: | TGGATGCTCTCATCAGGACAG |
| mouse DAP12-F: | AAGATGCGACTGTTCTTCCGT |
| mouse DAP12-R: | CCAGGGCAATCAGCAGAGTC |
| mouse Syk-F: | AGTAAGAGTGACGTTTGGAGC |
| mouse Syk-R: | CCAGCACAGGTTCATCAGGT |
| mouse MYH9-F: | ATCTCGTGCTATCCGCCAAG |
| mouse MYH9-R: | CGTTGGACAGGAAGCGGTAT |
| 3×DAP12-F： | CCCAAGCTTGCCACCATGGGAAGACTGGGGCCAT |
| 3×DAP12-R： | CCGCTCGAGTCACTTGTAGTACTGCCGCTGGGTA |
| DAP12(D50A)-F: | GGGGATCTGGTGCTGGCCCTCCTCATCGCCCTG |
| DAP12(D50A)-R: | CAGGGCGATGAGGAGGGCCAGCACCAGATCCCC |
| DAP12-Y86F-F: | ACAGAGTCTGCTTTCCAGGAGCTCCAA |
| DAP12-Y86F-R: | TTGGAGCTCCTGGAAAGCAGACTCTGT |
| DAP12-Y97F-F: | AGGTCAGATGTCTTCAGCGACCTCAAC |
| DAP12-Y97F-R: | GTTGAGGTCGCTGAAGACATCTGACCT |
| DAP12-(2Y-2F)-F： | AGGCAGTTTTTTAAATGAGGATCCCGGGTGGCA |
| DAP12-(2Y-2F)-R： | TGCCACCCGGGATCCTCATTTAAAAAACTGCCT |
| DAP12-(Y58-F)-F: | GCCCTGGCTGTGTTTTCCCTGGGTCGG |
| DAP12-(Y58-F)-R: | CCGACCCAGGGAAAACACAGCCAGGGC |
| DAP12-ΔICD-F: | CGGAATTCCAGAGAGAATGCAGCTGCT |
| DAP12-ΔICD-R: | GCTCTAGATCACACAGCCAGGGCGAT |
| DAP12-ΔECD-F: | CCCAAGCTTGGCATCCTGGCG |
| DAP12-ΔECD-R: | GCTCTAGATCATTTGTAATACTGCCTCTGTGTG |
| DAP12-ΔTM1-F: | GCTGCTCCGCCGTGAGCCCCCTGGGGGATCTGGTGCTGAC |
| DAP12-ΔTM1-R: | GTCAGCACCAGATCCCCCAGGGGGCTCACGGCGGAGCAGC |
| DAP12-ΔTM2-F: | GCATCCTGGCGGGGATCGTGCTCCTCATCGCCCTGGCTGT |
| DAP12-ΔTM2-R: | ACAGCCAGGGCGATGAGGAGCACGATCCCCGCCAGGATGC |
| DAP12-ΔTM3-F: | TGGGGGATCTGGTGCTGACCTACTCCCTGGGTCGGCTGGT |
| DAP12-ΔTM3-R: | ACCAGCCGACCCAGGGAGTAGGTCAGCACCAGATCCCCCA |
| DAP12-mRFP-infusion-F: | AGGGAGACCCAAGCTTATGGGGCGCCTCGGACCC |
| DAP12-mRFP-infusion-R: | CGTTACTAGTGGATCCTTTGTAATACTGCCTCTGTGTGTTG |
| Syk-myc-his-F: | CGGGATCCATGGCAGACAGTGCCA |
| Syk-myc-his-R: | CCGCTCGAGATTAACCACATCGTAGTAGTAATTG |
| EGFP-NMHC-IIA-F: | AGGGAGACCCAAGCTTATGGCACAGCAAGCTGCC |
| EGFP-NMHC-IIA-R: | CGTTACTAGTGGATCCTTCGGCAGGTTTGGCCTC |
| IIA-A-GST-Infusion-F: | GGGGCCCCTGGGATCCATGGCACAGCAAGCTGCC |
| IIA-A-GST-Infusion-R: | GATGCGGCCGCTCGAGCTAGAGGACACACGCCTGC |
| IIA- B-GST-Infusion-F: | GGGGCCCCTGGGATCCATGGTTAAGCCACTGCTGCAGG |
| IIA- B-GST-Infusion-R: | GATGCGGCCGCTCGAGCTAGTAGTCCTTCATCTGAGCCTGC |
| IIA- C-GST-Infusion-F: | GGGGCCCCTGGGATCCATGCGGGAGCTGGAGGAC |
| IIA- C-GST-Infusion-R: | GATGCGGCCGCTCGAGTTCGGCAGGTTTGGCCTC |
| IIA- A-Fc-Infusion-F: | CTTGTCACGAATTCGATGGCACAGCAAGCTGCC |
| IIA- A-Fc-Infusion-R: | GAGGACACACGCCTGCTTCCCGTCCATG |
| IIA- B-Fc-Infusion-F: | CAGGCGTGTGTCCTCATGGTTAAGCCACTGCTGC |
| IIA- B-Fc-Infusion-R: | GTAGTCCTTCATCTGAGCCTGCAGCTTCCGCAGC |
| NMHC-IIA-Fc-Infusion-R: | AGTTTTGTCAGATCTTTCGGCAGGTTTGGCCTCA |
